# Preferences for nutrients and sensory food qualities identify biological sources of economic values in monkeys

**DOI:** 10.1073/pnas.2101954118

**Published:** 2021-06-21

**Authors:** Fei-Yang Huang, Michael P. F. Sutcliffe, Fabian Grabenhorst

**Affiliations:** ^a^Department of Physiology, Development and Neuroscience, University of Cambridge, CB2 3DY Cambridge, United Kingdom;; ^b^Department of Engineering, University of Cambridge, CB2 1PZ Cambridge, United Kingdom;; ^c^Wellcome-MRC Institute of Metabolic Science, CB2 0QQ Cambridge, United Kingdom

**Keywords:** reward, decision-making, primate, ecology, obesity

## Abstract

Value is a foundational concept in reinforcement learning and economic choice theory. In these frameworks, individuals choose by assigning values to objects and learn by updating values with experience. These theories have been instrumental for revealing influences of probability, risk, and delay on choices. However, they do not explain how values are shaped by intrinsic properties of the choice objects themselves. Here, we investigated how economic value derives from the biologically critical components of foods: their nutrients and sensory qualities. When monkeys chose nutrient-defined liquids, they consistently preferred fat and sugar to low-nutrient alternatives. Rather than maximizing energy indiscriminately, they seemed to assign subjective values to specific nutrients, flexibly trading them against offered reward amounts. Nutrient–value functions accurately modeled these preferences, predicted choices across contexts, and accounted for individual differences. The monkeys’ preferences shifted their daily nutrient balance away from dietary reference points, contrary to ecological foraging models but resembling human suboptimal eating in free-choice situations. To identify the sensory basis of nutrient values, we developed engineering tools that measured food textures on biological surfaces, mimicking oral conditions. Subjective valuations of two key texture parameters—viscosity and sliding friction—explained the monkeys’ fat preferences, suggesting a texture-sensing mechanism for nutrient values. Extended reinforcement learning and choice models identified candidate neuronal mechanisms for nutrient-sensitive decision-making. These findings indicate that nutrients and food textures constitute critical reward components that shape economic values. Our nutrient-choice paradigm represents a promising tool for studying food–reward mechanisms in primates to better understand human-like eating behavior and obesity.

## Significance

Preferences for foods high in sugar and fat are near universal and major contributors to obesity. Additionally, human food choices are sophisticated and individualistic: we choose by evaluating a food’s nutrients and sensory features and trading them against quantity and cost. To understand the mechanisms behind human-like food choices, we developed an experimental paradigm in which monkeys chose nutrient rewards offered in varying quantities. Resembling human suboptimal eating, the monkeys’ fat and sugar preferences shifted their nutrient balance away from dietary reference points. Formally defined economic values for specific nutrients and food textures explained the monkeys’ preferences and individual differences. Our findings show how human-like preferences derive from biologically critical food components and open up investigations of underlying neural mechanisms.

The concept of “value” plays a fundamental role in behavioral theories that formalize learning and decision-making. Economic choice theory examines whether individuals behave as if they assigned subjective values to goods, which are inferred from observable choices (1, 2). In reinforcement learning, values integrate past reward experiences to guide future behavior (3, 4). Although these theories have been critical for revealing how choices depend on factors such as probability, risk, and delay (2, 4, 5), they do not explain how values and preferences are shaped by particular properties of the choice objects themselves. Why do we like chocolate, and why do some individuals like chocolate more than others? In classical economics, one famously does not argue about tastes (6). By contrast, biology conceptualizes choice objects as rewards with well-defined components that benefit survival and reproductive success and endow rewards with value (4). Here we followed this approach to investigate how the biologically critical, intrinsic properties of foods—their nutrients and sensory qualities—influence values inferred from behavioral choices and help explain individual differences in preference.

The reward value of food is commonly thought to derive from its nutrients and sensory properties: sugar and fat make foods attractive because of their sweet taste and rich mouthfeel. Sensory scientists and food engineers seek to uncover rules that link food composition to palatability (7–10). Similarly, ecological foraging theory links animals’ food choices to nutritional quality (11). By contrast, in behavioral and neuroscience experiments, food components are often only manipulated to elicit choice variation but rarely studied in their own right. Here, we aimed to empirically ground the value concept in the constitutive properties of food rewards. We combined a focus on specific nutrients and food qualities with well-controlled repeated-choice paradigms from behavioral neurophysiology and studied the choices of rhesus monkeys (*Macaca mulatta*) for nutrient-defined liquid rewards.

Like humans, macaques are experts in scrutinizing rewards for sophisticated, value-guided decision-making (4, 12–15). This behavioral complexity, the closeness of the macaque brain’s sensory and reward systems to those of humans (16), and the suitability for single-neuron recordings make macaques an important model for studying food–reward mechanisms with relevance to human eating behavior and obesity (17).

Previous studies in macaques uncovered key reward functions and their neuronal implementations, including the assignment of values to choice options (13, 18–25), reinforcement learning (4, 26) and reward-dependence on satiety and thirst (7, 27, 28). Despite these advances, behavioral principles for nutrient rewards in macaques remain largely uncharacterized. The typical diet of these primates includes a broad variety of foods and nutrient compositions (29, 30). Their natural feeding conditions require adaptation to both short-term and seasonal changes in nutrient availability and ecologically diverse habitats (31, 32). Thus, the macaque reward system should be specialized for flexible, nutrient-directed food choices. Accordingly, we manipulated the fat and sugar content of liquid food rewards to study their effects on macaques’ choices. We addressed several aims.

First, we tested whether macaques’ choices were sensitive to the nutrient composition of rewards, consistent with the assignment of subjective values. In previous studies, macaques showed subjective trade-offs between flavored liquid rewards (12, 13). We hypothesized that nutrients and nutrient-correlated sensory qualities constitute the intrinsic food properties that shape such preferences. We focused on macronutrients (carbohydrates, fats, and proteins), specifically sugar and fat, because of their relevance for human overeating and obesity, and their role in determining sensory food qualities. As nutrients are critical for survival and well-being, nonsated macaques should prefer foods high in nutrient content. In addition, like humans, they may individually prefer specific nutrients and sensory qualities (e.g., valuing isocaloric sweet taste over fat-like texture). Because nutrients are basic building blocks of foods, establishing an animal’s “nutrient–value function” could enable food choice predictions across contexts.

Second, to identify a physical, sensory basis for nutrient preferences, we developed engineering tools to measure nutrient-dependent food textures on biological surfaces that mimicked oral conditions. Although sugar is directly sensed by taste receptors (33), the mechanism for oral fat-sensing remains unclear. While the existence of a “fat taste” in primates is debated (34), substantial evidence points to a somatosensory, oral–texture mechanism (7, 9). Fat-like textures reliably elicit fatty, creamy mouthfeel (8) and activate neural sensory and reward systems in macaques (35) and humans (36, 37). Two distinct texture parameters are implicated in fat-sensing: viscosity and sliding friction, reflecting a food’s thickness and lubricating properties, respectively (38–40). We hypothesized that these parameters mediate the influence of fat content on choices.

Third, we compared the monkeys’ choices to ecologically relevant dietary reference points. In optimal foraging theory (41), animals maximize energy as a common currency for choices (“energy maximization”). Alternatively, animals may balance the intake of different nutrients (“nutrient balancing”) (42–44) or choose food based on the reward value of specific sensory and nutrient components (“nutrient reward”) (7, 45). We evaluated these strategies in a repeated-choice paradigm suited for neurophysiological recordings and derived hypotheses about the neuronal mechanisms for nutrient-sensitive decision-making (e.g., “energy-tracking neurons” versus “nutrient–value neurons”—*Discussion*).

Finally, based on our behavioral data, we explored in computational simulations how theories of reinforcement learning and economic choice can be extended by a nutrient–value function. Together with recently proposed homeostatic reinforcement learning (46), nutrient-specific model parameters may optimize predictions when choices depend on nutrient composition and homeostatic set-points.

## Results

### Nutrient–Choice Task and Nutrient–Reward Design.

**Fig. 1.**
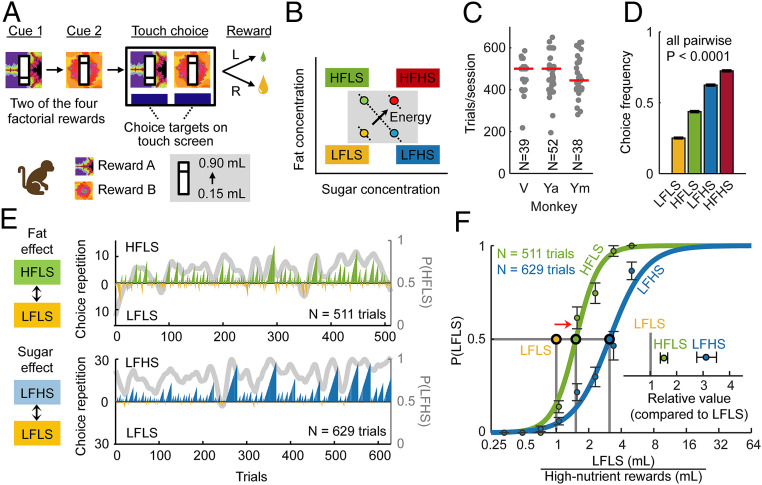
Choice task and reward design for studying nutrient influences on monkeys’ choices. (*A*) Nutrient–choice task. Monkeys chose from two sequentially presented options. Conditioned stimuli predicted different liquid rewards; magnitude bars predicted randomly varying reward amounts. (*B*) Nutrient–reward design. Liquid rewards differed in sugar and fat concentration. LFLS: low-fat, low-sugar; HFLS: high-fat, low-sugar; LFHS: low-fat, high-sugar; and HFHS: high-fat, high-sugar. Rewards were matched in flavor (peach of blackcurrant) and other ingredients (protein, salt, etc.); HFLS and LFHS were matched in energy content (isocaloric); HFHS had a higher energy content; and LFLS was lowest in energy content. (*C*) Completed choice trials per testing session in each animal (N: number of sessions). (*D*) Choice frequencies for each nutrient reward (± SEM), across sessions and animals (N = 55,205 trials). (*E*) Choice biases for fat and sugar in single sessions. Trial-by-trial choice records of two representative sessions from monkey Ya choosing between a low-nutrient option (yellow) and rewards with added fat (HFLS, green, *Top*) or sugar (LFHS, blue, *Bottom*). Upward/downward bars represent choices for high-/low-nutrient rewards; bar height indicates repeated choice counts. Gray curve shows choice frequency for high-nutrient rewards (seven-trial running average). (*F*) Nutrient–value functions. Choice frequencies for the low-nutrient reference as a function of offered magnitude ratio (LFLS/high-nutrient rewards ± SEM). Indifference points, estimated by inflection points of fitted sigmoid curves, identify relative values of the high-nutrient rewards, measured on the common scale of the low-nutrient reference. (*Inset*) Relative values of high-fat and high-sugar rewards and their 95% CIs.

Three rhesus monkeys performed in a binary choice task in which they repeatedly chose between varying amounts of dairy-based liquid rewards that differed in nutrient content (Fig. 1*A* and *SI Appendix*). Each testing day, two liquids were pseudorandomly selected from a 2 × 2 factorial design with fat and sugar level as factors (Fig. 1*B* and *SI Appendix*, Table S1): the low-fat low-sugar liquid (LFLS), the high-fat low-sugar liquid (HFLS), the low-fat high-sugar liquid (LFHS), and the high-fat high-sugar liquid (HFHS). The LFLS liquid was lowest in energy content; the HFLS and LFHS liquids were matched in energy content (isocaloric) at an intermediate level; and the HFHS liquid was highest in energy content. Liquid types were cued by conditioned visual stimuli; varying reward amounts were cued by pretrained magnitude bars. This design allowed us to test how fat, sugar, and their interactions influenced the monkeys’ choices. Importantly, the rewards were matched in juice flavor (peach or blackcurrant), temperature, and other ingredients (protein, salt, etc.). Systematic choice biases could therefore only be attributed to manipulated nutrient content and nutrient-related sensory qualities.

All three monkeys were motivated by the nutrient rewards to perform hundreds of choice trials each day (Fig. 1*C*). Across animals, choice frequencies for the different liquids indicated clear preferences for high-nutrient rewards (Fig. 1*D*): the HFHS liquid was most frequently chosen, the LFHS liquid was preferred over the HFLS liquid, and the LFLS liquid was least preferred. Thus, dairy-based, nutrient-defined liquids constituted potent rewards and the monkeys preferred liquids with high nutrient content.

### Preferences and Economic Values for Fat and Sugar Rewards: Example Data.

In the two sessions shown in Fig. 1*E*, monkey Ya chose between a low-nutrient liquid (LFLS) and liquids with either added fat (HFLS, top panel) or sugar (LFHS, bottom), all of which were peach flavored. The monkey preferred high-fat and high-sugar liquids by choosing them more frequently and more repeatedly, as indicated by repeated choice counts (“run lengths”). In both sessions, the monkey showed a marked choice bias for high-nutrient liquids (green, blue) over the low-nutrient liquid (yellow), resulting in higher choice frequencies than expected by chance (*P* < 10^−10^, binomial test) and higher repeated choice counts (*P* < 10^−10^, likelihood ratio test). To quantify the monkey’s willingness to trade reward magnitudes for fat and sugar (i.e., give up liquid to obtain nutrients), we calculated the percentage intake of nutrients and liquid amounts compared to the total offers in each session. The monkey gave up 8% of offered reward magnitudes to obtain an additional 33% of fat (compared to an agent who maximized reward magnitudes) and gave up 26% of offered magnitudes to obtain an additional 45% of sugar. These trade-offs suggested that the monkey assigned subjective, economic values to fat and sugar for making choices.

We constructed “nutrient–value functions” that related the ratio of offered liquid magnitudes to the monkey’s choice frequency to quantify subjective nutrient values (Fig. 1*F*). From fitted psychometric functions, we identified the magnitude ratio at which the monkey chose both options with equal probability. This “indifference point” revealed how many units of low-nutrient reward were equally preferred to one unit of high-nutrient reward; it thus measured value on the common scale of the low-nutrient reference. Indifference points were right-shifted from the point of objective equality (magnitude ratio = 1), indicating that more low-nutrient reward was required to compensate for its lower value compared to the high-nutrient rewards. Specifically, the monkey would choose the high-fat and low-nutrient liquids equally at a ratio of 1.52 (LFLS/HFLS). Thus, one unit of high-fat liquid was worth 1.52 units of low-nutrient liquid. The animal required even higher low-nutrient magnitudes when choosing against the high-sugar liquid. Small confidence intervals indicated precise estimates of indifference points (Fig. 1 *F*, *Inset*).

Thus, the monkey’s preferences systematically reflected trade-offs between reward amounts and nutrient compositions, consistent with the assignment of economic values to choice options.

### Nutrient–Value Functions for Fat and Sugar Rewards across Animals, Food Flavors, and Choice Tasks.

**Fig. 2.**
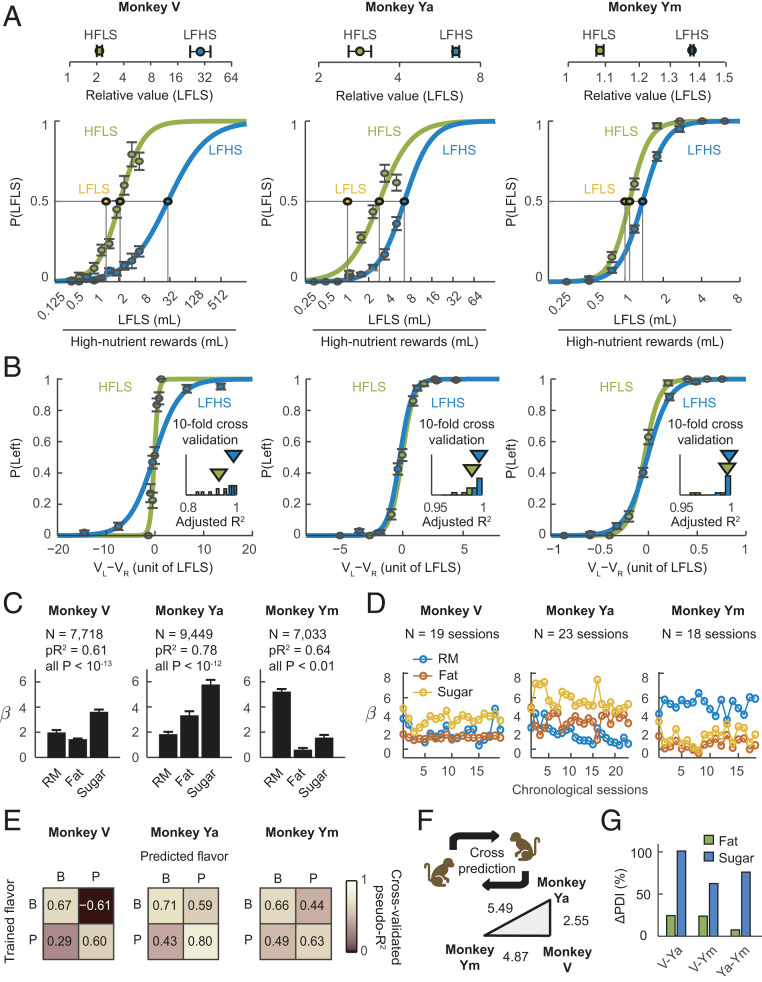
Nutrient–value functions explain monkeys’ fat and sugar preferences, predict choices across flavors, and account for individual differences. (*A*) Nutrient–value functions in three animals across sessions. Choice frequencies for the low-nutrient reference as a function of offered magnitude ratios (error bars: SEM). (*Top*) Indifference points identify animal-specific subjective values for high-nutrient rewards (relative values and 95% CIs). (*B*) Out-of-sample validation of subjective values. Relationships between choice probabilities and subjective value differences. Subjective values were derived from animal-specific indifference points in *A* by transforming offered magnitudes of high-nutrient rewards into value equivalents of the low-nutrient option (10-fold cross validation). (*Insets*) Adjusted R^2^ values from sigmoid fits. (*C*) Nutrient values across all choice conditions. Mixed-effects logistic regression of monkeys’ choices on reward magnitudes (RM) and fat and sugar contents (“nutrient model”), calculated over all sessions. All three animals showed significant coefficients (± SEM) for fat and sugar on choices. (*D*) Stable fat and sugar effects across testing sessions, suggested by chronological session-wise regression coefficients from mixed-effects nutrient model. (*E*) Cross-flavor choice prediction. Confusion matrices show cross-validated pseudo-R^2^ values obtained by fitting the nutrient regression on choices for one flavor (P: peach, B: blackcurrant) to predict choices for the other flavor. (*F*) Cross-animal choice prediction. For each animal pair, we used one monkey’s nutrient–value function to predict the other monkey’s choices. We defined a PDI based on the average log-likelihood ratio of mutual cross-animal predictions (shown as numbers in the triangle plot). Longer triangle side-lengths represent larger discrepancies between animals’ nutrient preferences. (*G*) Fat–sugar contributions in explaining individual differences. Percentage increases in PDIs after independently including fat or sugar regressors into the basic regression for pair-wise cross-animal predictions.

We found clear nutrient preferences in aggregated choice data across testing days and monkeys, despite individual differences. Choice frequencies, choice repetitions, and explicit nutrient–magnitude trade-offs indicated that the monkeys preferred the high-fat and high-sugar liquids to the low-nutrient reference (*SI Appendix*, Fig. S1). Nutrient–value functions showed that all three animals gave up more low-nutrient liquid in exchange for fat and sugar, with particularly strong trade-offs in monkeys V and Ya (Fig. 2*A*). Monkey V showed the strongest sugar preference, requiring up to 32 times the offered high-sugar reward amount to choose the low-nutrient liquid (in other words, one unit of the LFHS liquid was worth 32 units of the LFLS liquid). We validated these value estimates by transforming the trial-by-trial offered magnitudes of each high-nutrient reward into animal-specific value equivalents of the low-nutrient reference to predict the animal’s choices (out-of-sample prediction; *SI Appendix*). The resulting value differences in units of the low-nutrient reference predicted the animals’ choices well in all three monkeys, as indicated by orderly psychometric curves and model-fit statistics (Fig. 2*B*). Further analyses indicated that these values were robust and complied with transitivity (*SI Appendix*, Figs. S2 and S3).

We used mixed-effects multinomial logistic regression to estimate nutrient–value functions from the animals’ trial-by-trial choices across all stimuli while controlling for reward magnitudes, left–right biases, and random effects across testing sessions. Our main regression (“nutrient model;” *SI Appendix*, Tables S2 and S3) identified significant positive coefficients for fat and sugar in each animal (Fig. 2*C*) in addition to magnitude effects. Pseudo-R^2^ values indicated good model fits (*SI Appendix*, Table S2). By quantifying the subjective weight of specific nutrients on choices, the regression coefficients modeled each animal’s nutrient–value function. Regression coefficients for fat and sugar were stable in all animals (Fig. 2*D*). Notably, because each liquid type was cued by a new visual stimulus in each session, the observed stable nutrient effects that generalized across sessions were not explained by preferences for specific visual cues. Effects of recent nutrient choices on current-trial choices varied across animals but indicated that recent fat choices increased the likelihood of current-trial fat choice (positive feedback), whereas recent sugar choices reduced fat choices (negative cross-nutrient feedback, *SI Appendix*, Fig. S4).

In addition to nutrient content, food flavor is a key determinant of reward value (7, 47). Mixed-effects regression on a separate data set involving blackcurrant-flavored liquids showed significant fat effects in all three animals and significant sugar effects in two animals (*SI Appendix*, Table S3); the effect of sugar in animal V was captured by a significant fat–sugar interaction, likely indicating subjective flavor–nutrient interaction. To quantify cross-flavor generalization of nutrient preferences, we derived nutrient–value functions from logistic regressions for one flavor to predict choices for the other flavor. Model-fit statistics (cross-validated pseudo-R^2^) confirmed accurate cross-flavor predictions for two animals (Ya, Ym) and lower but above-chance accuracy for animal V (Fig. 2*E*). Thus, nutrient preferences for fat and sugar were largely consistent across juice flavors and could be predicted by an animal’s nutrient–value function.

The monkeys showed marked individual differences in their nutrient preferences. To quantify this distinctiveness of nutrient preferences, we used one monkey’s nutrient–value function to cross-predict another’s choices. For each animal pair, we defined a preference dissimilarity index (PDI) based on averaged log-likelihood ratios of mutual cross-animal predictions (*SI Appendix*). Using pairwise PDIs, we constructed a triangle whose side-lengths visualized discrepancies between animals’ nutrient preferences (Fig. 2*F*). This procedure showed that monkey Ym had the most distinct choice patterns, while monkeys V and Ya shared similar preferences, indicated by small PDI and good cross-predictions. Repeating the cross-predictions by including fat, sugar, or both nutrient regressors showed that fat and sugar accounted for the preference dissimilarities between animals, with sugar contributing three to five times more than fat (Fig. 2*G*). Thus, distinct nutrient valuations accounted for individual differences in preference.

We replicated and extended these findings in an additional experiment in which monkeys chose directly between all nutrient rewards, offered randomly in the same session at constant magnitudes (*SI Appendix*, Fig. S5). We also included diluted cream as a high-fat, low-sugar stimulus. Choice frequencies confirmed the preference rankings observed in our main task, and regressions confirmed significant sugar and fat influences on choices and individual differences (*SI Appendix*, Fig. S5).

Thus, by trading nutrients against reward quantities, the monkeys chose as if they assigned economic values to specific nutrients. These nutrient–value functions were stable within animals, generalized across flavors and choice contexts, and explained individual differences.

### Food Texture: Viscosity and Sliding Friction as a Physical Basis for Fat-Sensing.

**Fig. 3.**
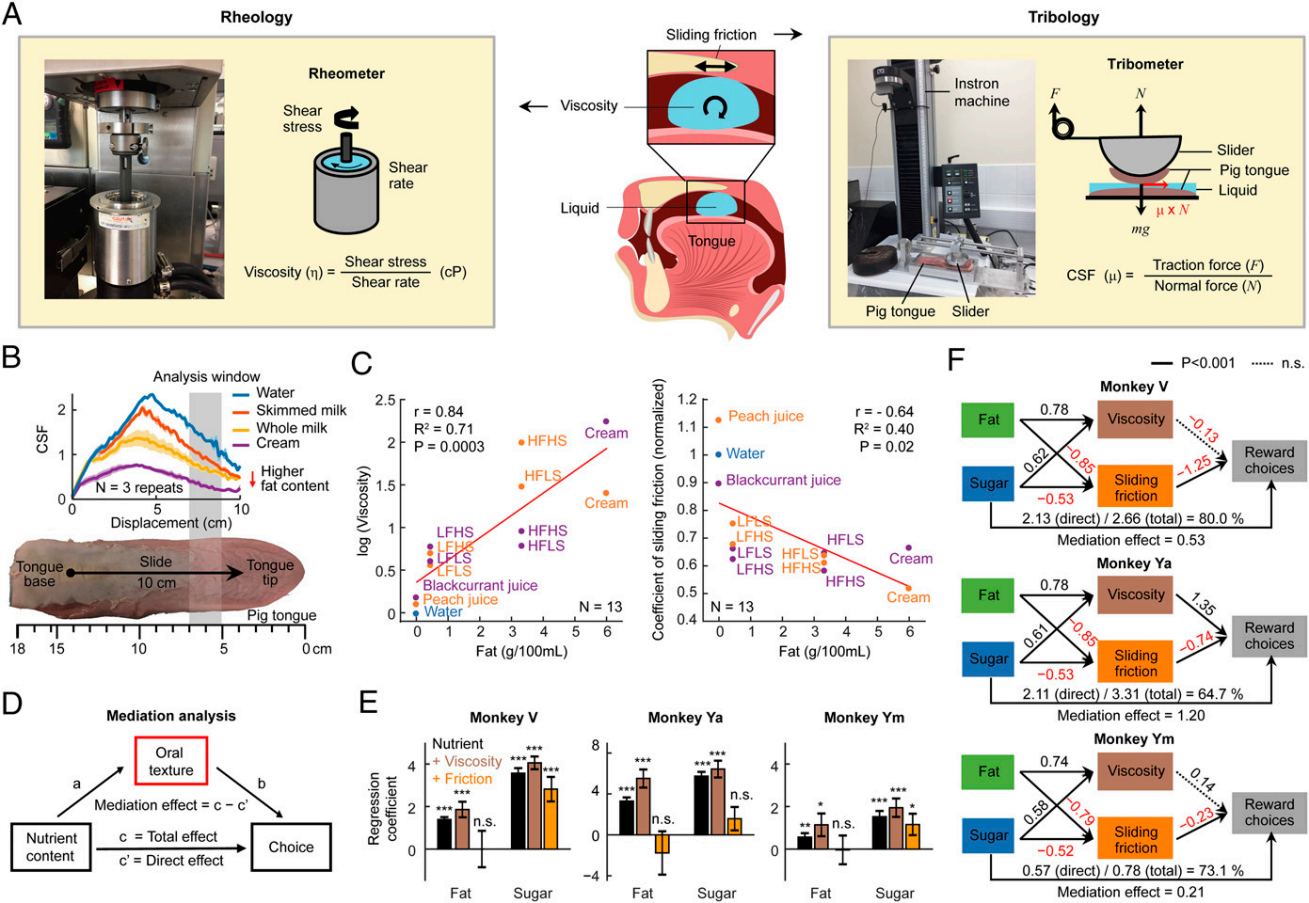
An oral texture-sensing mechanism mediates the influence of fat on choices. (*A*) Rheology and tribology measurements. Rheology examines liquid flow to characterize stimulus viscosity; tribology quantifies lubrication and friction between moving surfaces as the CSF. We measured viscosity by testing the liquids in a rotational rheometer (*Left*). We measured CSF using a custom-designed tribometer (*Right*) with biological tissue (fresh pig tongues) mimicking oral surfaces. We loaded 30 mL of testing liquid between pig tongues pulled by a slider controlled by an Instron Testing System from tongue base (posterior) toward tongue tip (anterior) at constant velocity (v = 16 mm/s). (*B*) Sliding friction measurements. Curves show CSFs for liquids with increasing fat content, measured from a single tongue (three repetitions per stimulus). CSF was normalized to the coefficients measured with water. (*C*) Viscosity and CSF as a function of fat content in our stimulus set for the two flavors (orange: peach and purple: blackcurrant; linear regressions). Measurements are shown for 13 stimuli: the four factorial stimuli with each of the two flavors, diluted cream with each flavor, diluted fruit juices, and water. (*D*) Mediation analysis. The aggregated influence of nutrient content on choices (total effects, *path c*) was decomposed into indirect effects mediated by texture (*path a*, *path b*) and direct effects (*path c’*). Mediation effects were significant if texture parameters replaced regression coefficients of the total effects ($c^{'}=c-b$). (*E*) Mediation effects of viscosity and CSF on the influences of fat and sugar on choices. Logistic regressions included fat and sugar regressors (“nutrient”) or additional viscosity (“+ Viscosity,” brown) or CSF (“+ Friction,” orange) regressors. (*F*) Path diagrams describing correlational relationships between nutrient content, texture parameters, and choices. Because the effect of fat on choices was fully accounted for by CSF (all animals) and viscosity (animal Ya), we included only the direct sugar effect. Significance of path coefficients derived from bootstrap (1,000 iterations).

So far, we have shown that the monkeys’ choices were guided by subjective evaluations of fat and sugar. These findings naturally raise the question of how the animals sensed the nutrient content of the liquids. To address this issue, we focused on the sensing of fat from food texture, as sugar can be directly sensed by taste receptors (33). Two specific texture parameters have been implicated in oral fat-sensing (Fig. 3*A*): viscosity and the coefficient of sliding friction (CSF) (8, 38–40). We hypothesized that these parameters mediated the influence of fat content on the monkeys’ choices. Testing this hypothesis required first measuring viscosity and CSF of our liquid rewards under biologically realistic conditions before relating these measures to choices using regression analysis.

We combined engineering tools from rheology and tribology to measure the viscosity and sliding friction of our nutrient rewards. The viscosity of a liquid is the resistance to motion against an applied force, defined as the quotient of shear stress and shear rate, commonly measured in a rotational rheometer (Fig. 3*A*) (39). By contrast, sliding friction measures the resistance to relative motion between contacting surfaces. This resistance depends on the loading force applied to the interface, the details of the contacting surfaces, and the properties of any lubricant between the surfaces (39). The CSF normalizes the friction force with the loading force to characterize the lubricating properties of a test liquid between contact surfaces. To approximate friction conditions in the oral cavity (i.e., the tongue sliding over the palate), we devised a tribometer using parallel-sliding pig tongues as biological surfaces rather than standard tribometers with circular-rotating glass and metal surfaces (Fig. 3 *A* and *B* and *SI Appendix*) (48). To mitigate inevitable biological tissue variations, we normalized CSF measurements to the CSF of the same tongue tissue lubricated with water before averaging measurements across tongues.

In validation tests, the CSF measurements reliably reflected increases in fat content in a series of fatty liquids (Fig. 3*B*). We next examined how viscosity and CSF were related to fat and sugar content in our nutrient-defined rewards. As expected, increased fat content correlated positively with viscosity and negatively with CSF (Fig. 3*C*). By contrast, sugar content had no significant correlations with either viscosity or CSF (*SI Appendix*, Fig. S6). To link these texture measurements to texture perceptions, we performed a psychophysical experiment in which human participants (*n* = 23) sampled the liquids used in the monkey experiments and rated subjective perceptions of the mouthfeel produced by the liquids (*SI Appendix*). We found that human psychophysical ratings of the thickness and oiliness of liquid rewards were positively related to stimulus viscosity and negatively related to CSF (all *P* < 0.002, multiple regression; *SI Appendix*, Fig. S6), suggesting that both texture properties contributed to texture sensations. Notably, oiliness was more strongly related to CSF than to viscosity (*P* = 0.011, *t* test on regression coefficients).

Thus, viscosity and sliding friction tracked the fat content of liquid nutrient rewards and produced distinct oral texture perceptions in humans. High-fat liquids were more viscous and slippery, which humans perceived as thick and oily mouthfeel, respectively.

### Oral Texture-Sensing Mediates the Influence of Fat on Choices.

To test whether oral texture parameters mediated the effect of nutrients on choices, we used structural equation modeling (SEM) based on a series of logistic regressions. We hypothesized that sugar was mainly sensed from sweetness (33) and exerted a direct, texture-independent influence on choices, whereas fat was indirectly sensed from viscosity and sliding friction (8, 40), which mediated the influence of fat on choices (Fig. 3*D*).

Regression-derived path coefficients within this framework supported our hypothesis (Fig. 3 *E* and *F*). As described above, viscosity and CSF correlated with fat but not sugar content. Although fat content itself had a significant effect on choice, this effect disappeared when texture parameters were included in the regression; specifically, CSF seemed to account fully for the effect of fat on choices (complete mediation effect, Fig. 3*E*). Thus, texture parameters accounted for the effect of fat on choices, suggesting oral texture as a critical intermediate mechanism that linked fat content to choices. By contrast, the effect of sugar on choices was largely unaffected by viscosity and CSF, confirming a direct, texture-independent influence. Across animals, CSF had particularly strong and robust influences on choice (Fig. 3*F*): negative and significant CSF path-coefficients indicated that the monkeys preferred liquids with a slippery oral texture typical of high fat content. By contrast, only monkey Ya had an additionally significant and positive effect of viscosity on choices. This result implied that whereas CSF mediated a common fat effect on choices, viscosity contributed to individual differences between animals.

We replicated and extended these findings in follow-up experiments involving choices between all rewards in the same session and an additional cream stimulus for wider nutrient and texture variation. In this independent data set, sugar again had a significant positive and direct effect on choice and the fat effect was accounted for by texture parameters (*SI Appendix*, Fig. S7). As in the main data set, CSF had a significant negative effect on choice in two animals; different from the main data set, viscosity had a significant positive effect on choice in all animals, likely due to the addition of cream as a preferred high-viscosity liquid.

These results indicated that the animals sensed fat content from viscosity and sliding friction and used this information for nutrient valuation and decision-making.

### Energy Maximization Does Not Explain the Influence of Nutrients on Choices.

According to optimal foraging theory, animals choose foods by maximizing energy intake as a common currency (i.e., calories per unit time) (41). We examined whether our animals’ choices were guided by subjective valuations of specific nutrients or by a behavioral strategy that maximized energy irrespective of nutrient composition.

**Fig. 4.**
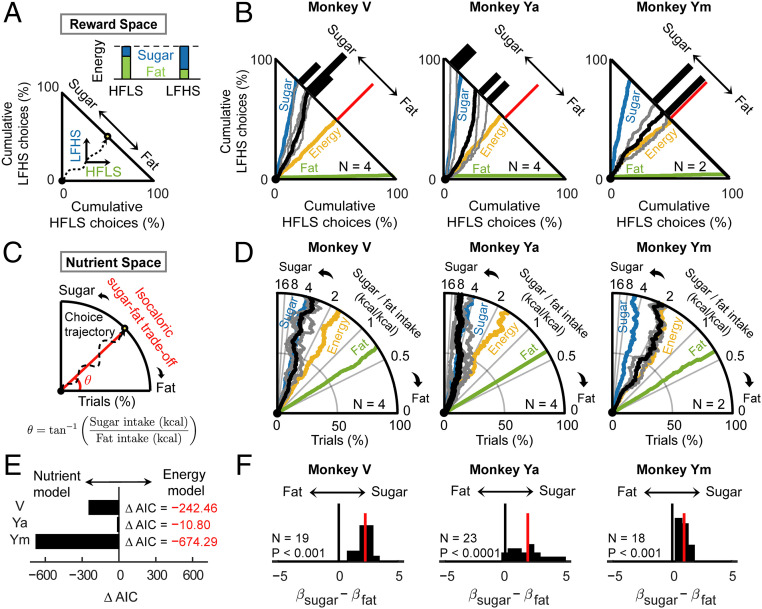
Energy maximization does not explain nutrient preferences. (*A*) Schematic of cumulative choice trajectory between isocaloric high-sugar (LFHS) and high-fat (HFLS) rewards in reward space. (*Inset*) Proportions of fat and sugar to matched energy content in the two rewards. (*B*) Cumulative choices between isocaloric high-sugar (LFHS) and high-fat (HFLS) rewards for the three animals (black: mean trajectory of actual choices, gray: single-session trajectories; colors: simulated choice trajectories based on reference strategies maximizing calories, fat, or sugar). All three monkeys’ choice trajectories were biased toward high-sugar reward. (*C*) Schematic of cumulative choices in nutrient space polar coordinates, showing dynamic fat–sugar trade-off (slope: relative fat–sugar intake ratio and radius: trial progression). (*D*) Choice trajectories transformed from reward space into nutrient space (same sessions as in *C*; black: actual choices; colors: reference simulated choices). (*E*) Model comparison based on Akaike Information Criteria (AIC) favored the nutrient model with separate fat and sugar regressors over the energy model in explaining reward choices. (*F*) Higher sensitivities to sugar compared to fat despite matched energy content suggested by differences in logistic-regression coefficients for isocaloric sugar and fat levels (*P* < 0.001, Wilcoxon signed-rank test).

We first focused on sessions in which monkeys chose between isocaloric high-fat and high-sugar rewards. Cumulative choice plots showed the animals’ dynamic, trial-by-trial choice trajectories and aggregated choice frequencies, which allowed comparisons between choice strategies based on nutrient preferences or energy maximization (Fig. 4*A*). Session-averaged choice trajectories showed that initially indiscriminate choices (when novel visual cues were introduced) gradually deviated toward the high-sugar reward to various extents in all three monkeys (Fig. 4*B*). Trajectories quickly deviated toward sugar in monkey Ya and monkey V, whereas monkey Ym’s trajectories initially followed energy maximization before deviating toward sugar. To directly examine the dynamics of fat and sugar intake, we transformed choice trajectories from reward space to nutrient space. We computed the ratio of fat to sugar intake for every choice and transformed the cumulative reward choices in Cartesian coordinates to angles in polar coordinates (Fig. 4 *C* and *D*); angles between curves and the horizontal axis thus represented the trade-off between fat and sugar. Distributing choices between reward options implied navigating the nutrient space by changing the fat-to-sugar intake ratio. Both monkey V and monkey Ya closely followed a sugar maximization strategy, while monkey Ym’s choices reflected a mixture between sugar and energy maximization. Additional tests involving isocaloric rewards with intermediate fat and sugar content confirmed a stronger preference for sugar, even when energy content was controlled (*SI Appendix*, Fig. S8).

We formally compared nutrient valuation and energy maximization choice strategies using logistic regressions on the full data set. We combined nutrient concentrations with reward magnitudes into a single energy content regressor (“energy model,” *SI Appendix*) and compared the energy model to the nutrient model with independent fat and sugar regressors. The nutrient model outperformed the energy model in explaining choices in all three monkeys (Fig. 4*E*). Further, as we set the high-fat and high-sugar level to be isocaloric, the normalized dichotomous fat and sugar regressors represented the same amount of additional calories. Therefore, the energy maximization strategy would predict equal regression coefficients for the isocaloric high-fat and high-sugar levels. By contrast, in all three monkeys, standardized regression coefficients for sugar were significantly higher than those for fat (Fig. 4*F*). Thus, monkeys did not equally value rewards with isocaloric fat and sugar content but preferred sugar over fat as source of energy.

Taken together, nutrient-biased choice trajectories, model comparisons, and differential sensitivities to isocaloric nutrients argued against strict energy maximization.

### Monkeys’ Preferences Shift Their Nutrient Balance from Dietary Reference Points.

**Fig. 5.**
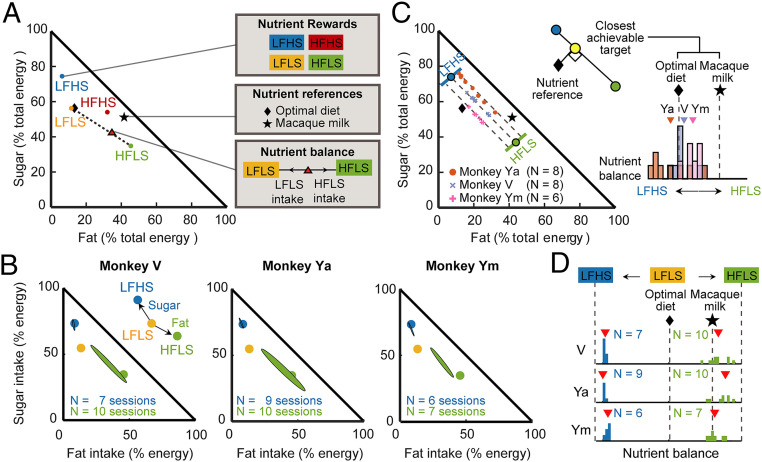
Monkeys’ preferences for fat and sugar shift their daily nutrient balance away from dietary reference points. (*A*) Schematic of a mixture triangle (49) that plots nutrient rewards, monkeys’ choices, and ecologically relevant reference points in a common space, defined by percentage proportions of fat, sugar, and protein to total energy. (Protein content was constant by design; therefore, the protein axis was similar across stimuli and unlabeled.) Colored circles: offered rewards; red triangle: nutrient balance resulting from monkey’s aggregated choices between LFLS and HFLS options (determined by the relative energy intake from each reward); and black markers: reference points. (*B*) Nutrient balances from monkeys’ aggregate choices occupied distinct and stable regions in nutrient space, depending on offered rewards. For choices between low-nutrient reward and high-fat or high-sugar options, nutrient balances were dominated by preferences for fat and sugar. Ellipses: 95% CIs. Inset shows how nutrient balance shifts away from LFLS reference toward high-nutrient stimuli. (*C*) Comparison with reference points: nutritionally optimal (low-fat, high-protein, and intermediate-carbohydrate) diet composition for adult macaques based on dietary guidelines (black diamond) and macaque milk (black star). Inset shows reference points projected onto the line connecting offered rewards (corresponding to the closest achievable approximations of reference points). (*D*) Monkeys deviated from optimal diet when choosing between high-fat, high-sugar rewards and a low-nutrient option. Histograms of nutrient balances in all three animals deviated from the projected reference point toward higher fat or sugar content (resulting in about 20% more fat or sugar intake than the optimal diet composition).

Balanced nutrient intake (i.e., maintaining stable proportions of nutrients) is crucial for health benefits and a choice strategy adopted by various animal species (42–44). Therefore, rather than only considering fat and sugar independently, we used the proportion-based Geometric Framework for Nutrition (44, 49) to model the relative fat and sugar intake in proportion of total consumed energy. We positioned our liquid rewards in a “mixer triangle” (49), based on their nutrient composition (percentage of total energy content, Fig. 5*A*). In the same space, the monkeys’ actual nutrient balances from their aggregated choices were linear interpolations between the two reward options, determined by the intake ratio of energy from each reward (Fig. 5*A*, red triangle). This model allowed us to examine whether the monkeys balanced nutrient intake or prioritized specific nutrients. We also compared the monkeys’ nutrient balances with ecologically relevant dietary reference points (Fig. 5*A*, black markers).

In the mixer triangle, the monkeys’ aggregate choices occupied distinct and stable regions depending on the offered rewards (Fig. 5*B*). Specifically, when choosing between the low-nutrient reward and the high-fat or high-sugar liquids, nutrient balances of all three monkeys were dominated by a preference for fat and sugar, respectively. Consequently, the proportions of energy intake from individual nutrients were on average 35% from fat and 45% from sugar (high-fat exposure) and about 5% from fat and 75% from sugar (high-sugar exposure), with greater variability during high-fat exposure (by design, protein accounted for ∼20% of total energy in all rewards). When offered nutritionally complementary rewards (LFHS and HFLS, Fig. 5*C*), about 15 to 25% of energy intake derived from fat and 55 to 65% from sugar. For other session types, energy intake derived to about 25 to 35% from fat and 45 to 60% from sugar. Thus, nutrient balance was not constant but varied with available food options, driven by a preference for high-sugar and high-fat rewards when they were available.

To appreciate the physiological implications of the observed nutrient balances, we compared them with two reference points, 1) a standard recommended diet composition for adult macaques, consisting of a low-fat, high-protein, and intermediate-carbohydrate diet (29), and 2) macaque milk, the exclusive nutrient source for infants (50). To determine whether the monkeys’ choices approximated these reference points, we projected each reference point onto the segment that connected the two available rewards in each choice condition (Fig. 5*C*). This segment defined the range of nutrient balances the monkeys could achieve by distributing their choices between the available rewards. When choosing between isocaloric high-fat and high-sugar rewards, nutrient balances clustered loosely around the optimal diet reference, with balances for monkeys Ya and Ym deviating toward higher sugar and fat intake, respectively (Fig. 5*C*). By contrast, when choosing between low-nutrient reward and high-fat or high-sugar rewards, all three animals deviated markedly from the projected reference point in the direction of higher fat or sugar content—they obtained ∼20% more energy from fat or sugar compared to a standard diet composition (Fig. 5*D*). Specifically, exposure to the high-sugar reward led to an increase of sugar intake of about 20% above reference, whereas exposure to the high-fat reward resulted in nutrient balance closer to the milk reference (Fig. 5*D*).

Thus, the monkeys’ preferences for high-sugar and high-fat rewards resulted in offer-dependent nutrient balances that deviated from dietary reference points.

### Nutrient-Sensitive Reinforcement Learning and Economic Choice Models.

Canonical behavioral frameworks such as reinforcement learning and economic choice theory do not specify how rewards with particular properties can differentially affect learning and choice. Based on the above findings, we examined whether a nutrient–value function could meaningfully extend such choice models to explain nutrient-sensitive choices.

**Fig. 6.**
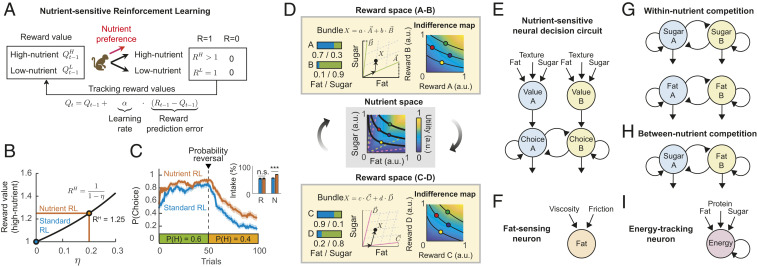
Nutrient-sensitive reinforcement learning, economic choice models, and candidate neuronal mechanisms. (*A*) A nutrient-sensitive reinforcement learning model updates values of choice options based on the nutrient content of reward outcomes. Depending on an agent’s nutrient–value function, high-nutrient rewards ($R^{H}>1$) elicit stronger value-updating than low-nutrient rewards ($R^{L}=1$). By comparison, a standard reinforcement model updates value irrespective of nutrient content $(R^{H}=R^{L}=1$). (*B*) Nutrient-sensitivity parameter η increments value for high-nutrient options (e.g., for η = 0.2, value increases from 1.0 to 1.25). (*C*) In a simulated reversal-learning task with high- and low-nutrient rewards (α=0.2, β=5,$R^{H}=1.25R^{L}$,N=100 trials), a nutrient-sensitive model learns faster to choose the high-nutrient option when it is associated with higher reward probability (P(H) = 0.6); following probability reversal (P(H) = 0.4), the nutrient-sensitive model switches more slowly to choosing low-nutrient reward than the standard model. (*Inset*) This learning pattern results in similar total reward outcomes (R) as the standard model but achieves higher nutrient intake (N). (*D*) Implications for economic choice theory. (*Top*) Indifference maps between combinations of rewards A and B describe an individual’s preferences between two reward options. (*Middle*) If choices depend on nutrient preferences, establishing indifference maps in nutrient space could predict preferences for untested rewards (*C* and *D*) of known nutrient composition (*Bottom*). (*E*–*I*) Neuronal hypotheses. (*E*) Nutrient-sensitive attractor-based decision circuit. Decision-making is implemented by competition through mutual inhibition between choice-coding neurons, biased by value inputs. Nutrient-sensitivity is implemented by fat, sugar, and texture inputs onto value neurons. (*F*) Neurons sensitive to dietary fat may receive separate viscosity and friction inputs. (*G*) Parallel circuits implementing decision-making based on sugar and fat content. (*H*) Decision circuit for nutrient prioritization. (*I*) Energy-tracking neurons integrate information across macronutrients.

Using simulations, we compared the performances of a Rescorla–Wagner model and an extended reinforcement learning model with a nutrient–value function (Fig. 6*A*). In a binary choice task, an agent chose between cues predicting high- and low-nutrient rewards with varying probabilities (*SI Appendix*, Fig. S9). By updating values solely based on past reward frequency, the standard RL model learned equally for both rewards regardless of nutrient composition. By contrast, the nutrient-sensitive model assigned higher value to high-nutrient reward outcomes, governed by a nutrient-sensitivity parameter (Fig. 6*B*). Accordingly, the nutrient-sensitive model learned faster when a high-nutrient reward was associated with high reward probability, which resulted in higher nutrient intake than the standard model at a similar reward rate (Fig. 6*C* and *SI Appendix*, Fig. S9). Thus, sensitivity to nutrient composition optimized learning performance when an agent’s value function prioritized specific nutrients.

In economic revealed-preference theory, indifference curves derived from choices connect multidimensional goods that differ in composition (e.g., fat–sugar content) but have the same level of satisfaction (utility) (1, 12). Multiple curves, each indicating a specific utility level, form an indifference map (Fig. 6*D*) that summarizes preferences to predict choices. The presently identified stable nutrient–value functions (Fig. 2*D*) imply that indifference maps can be defined in a common nutrient space to predict choices beyond specific tested foods. Accordingly, we examined how four representative reward bundles (A, B, C, and D) and their indifference relationships could be transformed from a tested reward (goods) space via a common fat–sugar nutrient space to an untested reward space. Specifically, points in the reward A-B space were linearly transformed to the underlying fat–sugar nutrient space with a nutrient–composition matrix and subsequently transformed to the novel reward space C-D using an inverse nutrient–composition matrix (Fig. 6*D*). Importantly, these transformations preserved the preference ranking of the reward bundles (A > B = C > D, indicated by stable bundle positions on indifference curves). This value-preserving property resulted from the underlying nutrient–value function that linked indifference analyses across different reward sets through their common ingredients. Supplementary analyses illustrated how indifference maps could be constructed from individual monkeys’ nutrient choices (*SI Appendix*, Fig. S10), although constructing complete nutrient–value functions would require more exhaustive tests.

These results suggest that incorporating nutrient-sensitivity in reinforcement learning and economic choice models can extend the predictive and explanatory validity of these models, especially when learning and choice depend on intrinsic reward components. Our data and simulations suggested candidate neuronal mechanisms for nutrient-sensitive decision-making (Fig. 6 *E*–*G*), on which we elaborate in *Discussion*.

## Discussion

We combined approaches from economics, food engineering, and ecology with a repeated-choice paradigm typical for neuroscience to study how formally defined economic values derive from a food’s nutrients and sensory qualities. We found that fat, sugar, and nutrient-correlated food textures influenced choices in two ways. First, they increased economic value in a manner that generalized across animals: the monkeys preferred fat, sugar, and related oral textures to low-nutrient alternatives. Second, they explained individual differences in the animals’ choices: each monkey showed an idiosyncratic trade-off between nutrients and reward quantities, consistent with the assignment of subjective values to choice options. Economic nutrient values were internally consistent, as they predicted choices across sessions, conditioned stimuli and food flavors, and were replicated in a separate experiment with more varied rewards. Thus, stable nutrient–value functions shaped the monkeys’ preferences. Cross-animal choice predictions succeeded for animals with comparable nutrient–value functions; nutrient and texture valuations accounted for individual differences. These effects were not explained by preferences for conditioned stimuli (which varied daily), other food properties (which we controlled), or task variables (*SI Appendix*, Table S3). Although object properties alone cannot fully explain reward functions and must be combined with behavioral assessment (4), our findings suggest that nutrients and food textures constitute biologically critical sources of economic values.

Previous studies showed that decision parameters including probability, risk, delay, and effort elicit subjective values in macaques (4, 21, 26, 51). Here, we held these factors constant and instead systematically varied the intrinsic, constituent properties of the reward itself. To appreciate this difference, consider a macaque pursuing a valued reward, such as a pomegranate. The fruit’s peel and arils contain fiber, sugar, protein, vitamins, and minerals, and its seeds are a source of fat. Together with the fruit’s sensory qualities, specifically its sweet taste and juicy texture, these object-defining intrinsic properties provide a physical basis for subjective reward values. By contrast, extrinsic factors, such as the probability, risk, effort, and delay associated with obtaining the fruit vary arbitrarily with the animal’s location relative to the reward. The distinction between intrinsic reward properties and external choice parameters is conceptually important because intrinsic reward properties are processed by dedicated neural and decision systems and are targeted by homeostatic regulation (45). For example, orbitofrontal cortex lesions impair macaques’ ability to adjust their food choices after food-specific satiation, which requires assessing the food’s intrinsic properties, but do not impair the tracking of stimulus-reward probabilities (52). Accordingly, we propose that sensitivities to nutrients and sensory food qualities constitute critical features of an animal’s value function, with implications for learning and choice theories, as discussed below.

Padoa-Schioppa and colleagues showed that macaques flexibly choose between different liquid rewards offered in varying amounts and that subjective values underlying these choices are explicitly encoded by neurons in orbitofrontal cortex and amygdala (13, 19). These brain structures are also essential for processing the current value of foods as influenced by satiation and for linking arbitrary visual stimuli to those values (14, 53). Other studies examined how thirst influences monkeys’ risk preferences (27) and the trading of reward bundles formalized by indifference maps (12). Building on these pioneering studies, our data identify specific nutrients and food textures as the sources of such subjective trade-offs in monkeys’ choices. Because nutrients and sensory properties are elementary building blocks of foods, establishing an animal’s nutrient–value function would enable choice predictions across foods and contexts (Fig. 6*D*) and may help uncover tuning properties of reward neurons (Fig. 6*E*). Notably, our study used nutrient-defined liquid rewards to allow for precise texture quantification and controlled delivery in neurophysiological recordings. Future studies could examine subjective values for properties of solid foods, including dietary fiber and crispy textures.

Supporting these notions, each monkey’s preferences were captured by a stable nutrient–value function that described how the animal weighed nutrient and sensory properties for decision-making (Fig. 2 *A*–*D*). This function predicted choices across different foods and naturally extended the value function of reinforcement learning models to nutrient rewards. We showed that incorporating nutrient-sensitivity could optimize reinforcement learning in situations when nutrients are behaviorally relevant, as in specific deficit states (Fig. 6*C*). Future extensions could explore how nutrient-specific values depend on changing physiological states (46).

Two key food texture parameters—viscosity and sliding friction—accounted for the effect of fat content on the monkeys’ choices (Fig. 3). In previous studies, human food ratings reflected these two properties in distinct ways (9, 40), but their impact on choices in a formal behavioral framework was not studied. We found that lower sliding friction of the liquid rewards was associated with increased choice. Thus, the animals preferred foods with a smooth, oily texture typical of high-fat stimuli. By contrast, the contribution of viscosity to fat preferences varied across animals. Together, subjective valuations of viscosity and sliding friction mediated the influence of fat content on choices and explained individual differences in food preferences. These results identify an oral texture-sensing mechanism as a sensory basis for nutrient values. Notably, distinct neurons in primate orbitofrontal cortex and amygdala encode oral viscosity and sliding friction (35), which raises the intriguing possibility that these neurons underlie the presently observed behavioral preferences.

We used the mixer triangle tool of the proportion-based Nutritional Geometric Framework (44, 49) to examine nutrient balances resulting from the monkeys’ choices. One influential view in ecology suggests energy as a common currency that animals maximize (41). More recent studies identified nutrient-balancing as a strategy adopted by various animals in the wild (42–44, 49). In our experiments, nutrient balances from monkeys’ choices were strongly offer dependent (Fig. 5). The animals chose primarily high-fat and high-sugar liquids when these were offered, suggesting a “nutrient–reward strategy,” rather than trading nutrients interchangeably in order to maintain a constant energy intake (“equal distance regulation”) (43). As a consequence, nutrient balances deviated from dietary reference points—the animals obtained higher sugar and fat content than recommended by dietary guidelines (29). This pattern of eating behavior resembles human overeating in the presence of high-fat and high-sugar foods (54). By combining our paradigm with neurophysiological recordings, future studies could help identify neuronal activities that underlie choice patterns leading to suboptimal nutrient balances.

Previous studies in humans documented hedonic liking for sugar, fat, and their interactions (55) but did not study preferences in the context of formal repeated-choice paradigms as we did here. Importantly, in our experimental paradigm, the monkeys made food choices in the absence of nutrient challenges or deficit states, as the animals received a standard daily diet that was nutritionally balanced (*SI Appendix*). Accordingly, the fat and sugar preferences reported here may differ from those of wild macaques and resemble more closely eating phenotypes observed in human free-choice scenarios. Under conditions of scarcity, macaques would likely adjust their choice strategy to counter deficit states. Thus, we expect nutrient–value functions to be state dependent.

Our behavioral and modeling results suggested candidate neuronal mechanisms for nutrient-sensitive decision-making that can be tested with single-neuron recordings (Fig. 6 *E*–*I*). An influential attractor neural network model implements decision-making as competition between choice-coding neurons, biased by value inputs (56). Our findings imply that value-coding neurons integrate fat, sugar, texture, and possibly other food components (e.g., protein, flavor, and temperature) to a subjective value signal (Fig. 6*E*). Our finding that texture properties mediated the effect of fat on choices (Fig. 3*E*) could suggest that fat-sensing neurons themselves receive separate inputs from viscosity and sliding friction neurons (Fig. 6*F*). Indeed, a recent study (35) reported such neurons in the orbitofrontal cortex (area 12), amygdala, and anterior insula; the study measured sliding friction with a steel ball rotating on a silicone disk. Our data and previous observations indicate that animals prioritize specific nutrients, for example in nutrient-deficit states (11). Thus, it could be computationally efficient to directly compare options in terms of specific nutrients (Fig. 6*G*). Further, offer-dependent nutrient balances (Fig. 5), preliminary evidence of nutrient history effects on choices (*SI Appendix*, Fig. S4), and ecological observations of nutrient-balancing (43) imply that neural circuits also implement between-nutrient competition (Fig. 6*H*) to fine-tune behavior to changing internal states. Finally, although monkeys did not strictly maximize energy (Fig. 4), we hypothesize that some neurons translate nutrients onto a common energy scale. Such energy-tracking neurons (Fig. 6*I*) could prevent overeating by negative postingestive feedback (47). The orbitofrontal cortex and amygdala participate in both decision-making and food evaluation (13, 15, 25, 35) and thus constitute ideal targets for testing these hypotheses experimentally.

Taken together, our data indicate that nutrients and sensory food qualities constitute biological sources of economic value that shape monkeys’ preferences. Food choices for high-fat and high-sugar rewards in our monkeys deviated from dietary reference points, resembling human-like suboptimal eating. Thus, our experimental nutrient-choice paradigm could serve as a tool to study behavioral and neuronal mechanisms underlying primate suboptimal food intake, to complement genetic (57) and neural-circuit approaches (47, 58) in other species. The similarity of the macaque brain’s sensory and reward systems to those of humans, their sophisticated human-like food choices, and their suitability for single-neuron recordings make macaques a promising model for studying food-reward mechanisms to better understand human eating behavior and obesity.

## Methods

### Animals.

Three adult male rhesus macaques (*M. mulatta*) participated in the present experiments: monkey Ya (weight during the experiments: 14 to 19 kg, age: 4 to 6 y), monkey V (weight: 9 to 11 kg, age 8 to 10 y), and monkey Ym (10 to 13 kg, age 4 to 6 y). The animals had free access to their standard diet before and after the experiments and received their main liquid intake in the laboratory (*SI Appendix*). The animals were on a standard diet for laboratory macaques. All animal procedures conformed to US NIH Guidelines and were approved by the Home Office of the United Kingdom.

### Nutrient Rewards.

We used commercial skimmed milk and whole milk (British skimmed milk and British whole milk, Sainsbury’s Supermarkets Ltd.) as baseline low-fat and high-fat liquids and flavored the liquids with fruit juice to increase palatability (*SI Appendix*, Table S1).

### Rheology and Tribology.

Rheology was performed using a Rheometric Scientific ARES controlled strain rheometer (TA Instruments). We measured the CSF at the Department of Engineering, University of Cambridge (*SI Appendix*). To reflect realistic lubrication conditions in the oral cavity, we devised a custom-designed tribometer using pig tongues as biological contacting surfaces (Fig. 3 *A*, *Right*).

### Data Analysis and Statistical Methods.

Unless otherwise specified, all data were analyzed separately for each animal and different juice flavors (peach and blackcurrant) using custom code and in-built functions in MATLAB R2017b. We adopted mixed-effects multinomial logistic regression analysis (*fitglme* function, MATLAB) to model the animals’ trial-by-trial choices.

### Geometric Framework for Nutrition.

We used the right-angled mixer triangle developed by Raubenheimer which implements a proportion-based variant of the Geometric Framework for Nutrition (44, 49) in which the available food compositions, reference nutritional targets, and the actual nutrient intake balance could be analyzed in a common framework (Fig. 5*A*). The compositions of food rewards were plotted in a mixer triangle (49) based on the percentage contribution of fat and sugar to total energy content.

## Supplementary Material

Supplementary File

## Data Availability

Behavioral data have been deposited in the GitHub repository (https://github.com/ckoceanaut/pnas2021_MonkeyNutrientChoices).
