# Supplementary material for: Preferences for nutrients and sensory food qualities identify biological sources of economic values in monkeys
Source: Proc Natl Acad Sci U S A. 2021 Jun 21;118(26):e2101954118. doi: 10.1073/pnas.2101954118 (PMC8255786; doi:10.1073/pnas.2101954118)
Supplement: Supplementary File [file pnas.2101954118.sapp.pdf]

PNAS
www.pnas.org

Supplementary Information for
Preferences for nutrients and sensory food qualities identify biological sources of economic values in monkeys

Fei-Yang Huang, Michael P. F. Sutcliffe and Fabian Grabenhorst

Corresponding author: Fabian Grabenhorst
Email: fabian.grabenhorst@gmail.com

This PDF file includes:

Supplementary text
Figures S1 to S10
Tables S1 to S3
SI References

Supplementary Information Text

METHODS

Animals. Three adult male rhesus macaques (*Macaca mulatta*) participated in the present experiments: monkey Ya (weight during the experiments: 14-19 kg, age: 4-6 years), monkey V (weight: 9-11 kg, age 8-10 years), and monkey Ym (10-13 kg, age 4-6 years). No statistical methods were used to predetermine sample size. The animals were trained and tested approximately one to two hours per day and five days per week over two years. We offered dairy-based nutrient rewards to the animals initially as additional treats and gradually as their main source of liquid intake during the behavioral tasks, supplemented by water outside of the tasks. On a given testing day, the animals had free access to their standard diet before and after the experiments and received their main liquid intake in the laboratory. The animals were on a standard diet for laboratory macaques, composed of high-protein dry pellets (% calories provided by protein: 30.36%, fat: 13.29%, carbohydrates: 56.34%), dried fruits, seeds, nuts, and fresh fruits and vegetables. We closely monitored the monkeys' general health conditions and body weights at all times to ensure their welfare after introducing the high-calorie rewards. No effects of the diet on the animals' health were observed. The animals' body weights were stable (V) or increased as expected for growing animals (Ya, Ym). As shown in Fig. 2D, nutrient preferences were relatively stable over time within individual animals. We found no clear relationships between body weight and these nutrient preferences; however, as our study was not specifically designed to test for such relationships we are cautious in interpreting this result.

All animal procedures conformed to US National Institutes of Health Guidelines. The experiments have been regulated, ethically reviewed and supervised by the following UK and University of Cambridge (UCam) institutions and individuals: UK Home Office, implementing the Animals (Scientific Procedures) Act 1986, Amendment Regulations 2012, and represented by the local UK Home Office Inspector; UK Animals in Science Committee; UCam Animal Welfare and Ethical Review Body (AWERB); UK National Centre for Replacement, Refinement and Reduction of Animal Experiments (NC3Rs); UCam Biomedical Service (UBS) Certificate Holder; UCam Welfare Officer; UCam Governance and Strategy Committee; UCam Named Veterinary Surgeon (NVS); UCam Named Animal Care and Welfare Officer (NACWO).

Behavioral tasks

Main choice task. In the main choice task (**Fig. 1A**), the monkey was seated in a custom-designed primate chair (Crist Instrument, Inc., Co., USA) in front of a horizontal touch monitor (EloTouch 1522L 15', Elo Touch Solutions, Inc., USA) with his mouth close to a spout that was connected to computer-controlled solenoid valves. A choice trial was initiated once the monkey made contact with an immobile touch-sensitive key. Two visual cues were sequentially presented on the monitor with overlaid reward-magnitude bars and then displayed in left-right arrangement, determined by random alternation. Each visual cue was associated with a fixed reward type in the same testing session. The chosen reward was immediately delivered to the monkey after each choice by which the animals learned the associations between conditioned stimuli and reward types at the beginning of each testing session. Pre-trained magnitude bars cued the randomized reward amount in each trial (0.15 - 0.90 mL per delivery). Higher magnitude bars signaled larger liquid amounts. After the monkey made a choice by touching one of two targets on the screen, displayed below the choice options, the exact amount of the chosen reward was delivered through the spout to the monkey. Trials were aborted in case of premature release of the touch key (before the choice epoch) or delayed choice responses (> 1,500 ms after the target presentation). The behavioral task and choice responses were controlled and registered by custom MATLAB codes (MATLAB version R2013b, The MathWorks, Inc., USA) using Psychophysics Toolbox version 3.0.8. The experimental set-up was interfaced with data acquisition boards (NI 6225; National Instruments Co., USA) installed on personal computers using Microsoft Windows 7/10 systems.

Alternative choice task. In the alternative choice task (**Fig. S5**), we offered all reward combinations within a single session for comparison and held the reward magnitudes

constant (0.3 mL). Specifically, the monkey was again sequentially presented with two options, this time each with three identical pre-trained visual cues that signaled the reward types. To simultaneously deliver a wider range of stimuli, we constructed an eight-channel delivery system that linked computer-controlled peristaltic pumps (ISM4408 Reglo ICC Digital four-channel, eight-roller peristaltic pump, Ismatec, Germany) to a custom-made eight-channel mouthpiece (Cambridge Electronics Workshop, Department of Experimental Psychology, University of Cambridge). The peristaltic pumps functioned at 100 revolutions per minute (RPM) and the activation duration at 0.4 s, which delivered approximately 0.3 mL of liquid for each activation. In addition to the four factorial stimuli, we included a low-sugar cream stimulus to introduce additional variation in nutrient content and food textures. Additional trial types involving choices with a broader stimulus set were not examined in the present manuscript.

Nutrient rewards. We prepared dairy-based nutrient-defined liquid rewards with 2 × 2 fat and sugar levels (**Fig 1B**), including low-fat low-sugar (LFLS), high-fat low-sugar (HFLS), low-fat high-sugar (LFHS) and high-fat high-sugar (HFHS) rewards. We used commercial skimmed milk and whole milk (British skimmed milk and British whole milk, Sainsbury's Supermarkets Ltd., UK) as baseline low-fat and high-fat liquids and flavored the liquids with fruit juice to increase palatability (peach juice, Robinsons Fruit & Barley Peach Squash, Robinsons Soft Drinks Ltd, UK; blackcurrant juice, Ribena© Blackcurrant Squash, Lucozade Ribena Suntory Ltd, UK). We calculated the nutrient content from the nutrient composition of these commercial products and developed a recipe for nutrient rewards with controlled fat and sugar content (**Table S1**). Specifically, we based on the fat content of the milk and adjusted the sugar content using white caster sugar (Sainsbury's White Caster Sugar, Sainsbury's Supermarkets Ltd., UK) to prepare the high-nutrient rewards. We added sugar to the whole milk to prepare the HFLS stimulus with equal sugar content to the LFLS stimulus but at higher fat level. For the low-fat high-sugar (LFHS) reward, we titrated the sugar content of the skimmed milk until the calorie content of the added sugar was identical to the calorie difference between HFLS and LFLS (27.2 kcal/100 mL = 6.8 g sugar/100 mL). We further added sugar to the whole milk up to the exact higher sugar level for HFHS. By keeping LFHS and HFLS isocaloric, we normalized the additional fat and sugar to their contained calories. In this design, fat and sugar levels were systemically manipulated while protein, salt, juice flavor and temperature were tightly controlled. The flavored cream used in the alternative choice task was a mixture of single cream (Sainsbury's British Single Cream 300 mL, Sainsbury's Supermarkets Ltd., UK), fruit juice and water. All rewards were prepared in 300 mL and stored under 4°C for 24 hours before the experiment. We monitored the temperature during the experiment using an infrared thermometer (Etekcity Lasergrip 1080 Non-contact Digital Laser IR Infrared Thermometer, Etekcity Co., USA) and offered the liquids to the monkeys at the temperature of around 17 ± 2°C.

Measuring oral texture parameters

Viscosity. We measured the viscosity of our liquid rewards at the Rheology Centre of the Department of Chemical Engineering, University of Cambridge. Measurements were performed using a Rheometric Scientific ARES controlled strain rheometer (TA Instruments, USA) with Couette geometry as follows: cup diameter = 34.0 mm, bob diameter = 32.0 mm, bob length = 34.0 mm (**Fig. 3A**, Left). The viscosity was determined by carrying out shear rate sweeps from 100 to 0.1 s^{-1} or 1 s^{-1} (reverse sweep) after allowing the samples to equilibrate at the experimental temperature for 300 seconds. Because viscosity was sensitive to temperature, we measured the samples at 18.5 ºC (delivered temperature), 25 ºC, and 37.5 ºC (body temperature) and used the measurement at 18.5 ºC for analyses based on the temperature of the testing liquids (17 ± 2°C) measured during the experiment. Once the sample reached the experimental temperature, the cup rotated first clockwise and then anticlockwise (two ways). In the time-sweeping measurement, the cup was rotated in a clockwise direction and the shear rate was fixed at 50 s^{-1}, at which rheological properties of liquid stimuli have been shown to be related to oral viscosity evaluations (1).

Coefficient of sliding friction (CSF). We measured the CSF for our liquid rewards at the Department of Engineering, University of Cambridge. To reflect realistic lubrication conditions in the oral cavity, we devised a custom-designed tribometer using pig tongues as biological

contacting surfaces (**Fig. 3A**, right). Our design included a flat aluminium platform to hold the nominally flat fixed base pig tongue. The upper moving tongue-tip tissue was attached using superglue onto a dome-shaped slider of radius 100 mm. Thus, the two tissues contacted with a nominal point contact, avoiding issues with alignment during the sliding process. The anatomically upper surface of each tongue was used for each of the two contacting surfaces. The dome-shaped slider was mounted using low-friction bushings onto a track containing two rails, pivoted at one end. A counterweight was used to balance out the weight of the rail elements of the track. Thus, the load through the contact between the tongues was only the weight of the dome-shaped slider and tongue tip specimen (2.58 ± 0.07 N), which did not vary as the slider moved along the track. The slider was attached via a light string and pulley to an Instron 5544 Universal Testing machine (Instron, USA). Preliminary tests confirmed that pulley friction was negligible. During testing, the moving tissue was loaded against the fixed base pig tongue with the testing liquids interfaced as lubricating layers. The Instron machine then imposed a fixed velocity (v = 16 mm/s) to the slider, while measuring the traction force using a load cell attached to the Instron machine. This design measured the sliding friction between the liquids and oral tissues to approximate the oral sensing conditions of the animals. Because we maintained a constant velocity during the test, according to Newton's First Law of Motion,

$$F - \mu N = 0$$

$$\mu = \frac{\text{traction force } (F)}{\text{loading force } (N)} = \frac{\text{traction force measured by the tensile machine}}{\text{total weight of the slider and the fixed tongue tip}}$$

where F is the applied force (traction) by the Instron machine, N is the loading force perpendicular to the contact surface (normal force), and μ, the coefficient of sliding friction, is the ratio of the traction force and the perpendicular loading force.

The day before testing, fresh pig tongues were obtained intact from a local butcher (Leech & Sons, Royston, UK) and were gently rinsed with water to remove residual blood and tissue fluids. We then retrieved the superficial 1 cm-thick, anterior 18 cm of the tongues for a flat contact surface that fitted onto the testing platform. The processed pig tongue slices were preserved in isotonic saline buffer (Phosphate-buffered saline, PBS, 1X, pH 7.4) in a freezer under 4 ºC overnight. On the testing day, we first prepared the contact surfaces by gluing one 18-cm tongue on the base platform and another tongue tip (5 cm) on the surface of the dome-shaped slider. We weighed the dome-shaped slider with the attached tongue tip to give the loading force for later calculation of the CSF. Before each measurement, we rinsed both tongue surfaces with 10 mL of isotonic saline buffer (PBS) three times to remove residual testing liquids and hydrate the tongue surfaces. Next, we loaded 30 mL of the testing liquids between the pig tongues and pulled the slider from the posterior tongue bases forward to the anterior tongue tip (**Fig. 3B**). All procedures were approved by the Departmental Safety Office, Department of Engineering, University of Cambridge, including Control of Substances Hazardous to Health (COSHH) and biohazard risk assessments.

For the formal testing of the stimuli, we measured each liquid with triplicate repeats using two pairs of pig tongues in opposite measuring orders to cancel out possible carryover effects (low-fat to high-fat liquids and reverse). We first averaged the triplicate measurements for each liquid and divided them by the corresponding loading force to obtain the coefficients of sliding friction along the tongue surface. We selected the anterior 5-7 cm of the tongues as the analysis window because the mechanosensory receptors are located mainly within the anterior two thirds of tongue (2, 3), and further anterior tongue tips were too thin for stable measurements. Because inevitable variations in tongue conditions influenced the absolute sliding-friction measurement, for each pair of testing tongues we normalized the measured coefficients with the coefficient of water obtained from the same pair of tongues. This normalization adjusted the offset in absolute measurement due to variations of the tongues and provided comparable results between different tongue pairs. Finally, we averaged the two normalized coefficients obtained from the different tongues for further analyses.

Our engineering approach measured sliding friction on biological tissues to approximate oral food-sensing conditions. We followed an earlier study that used pig tongues to measure sliding friction and link human fat perception to lower friction coefficients (4). The physical mechanism by which fat in emulsions lowers friction, i.e. produces lubrication, likely involves coalescence of fat droplets on oral surfaces that form an adhering fat layer (5-7). The present

study is the first to combine food-engineering methods with a controlled repeated-choice paradigm. To refine this technique, future studies should examine differences in hydrodynamic conditions between the tribological set-up and oral conditions, dependences on lubrication by saliva and testing speed, and measure related food properties (e.g. coalescence of fat particles, fatty-acid concentration) (8).

Data analysis and statistical methods

Unless otherwise specified, all data were analyzed separately for each animal and different juice flavors (peach and blackcurrant) using custom code and in-built functions in MATLAB R2017b.

Nutrient-specific choice biases. We assessed the preferences for fat and sugar content based on choice biases including choice frequency, choice repetition, magnitude-nutrient trade-off, and reward values between nutrient-defined rewards. Analyses were performed separately for each monkey.

Choice frequency*.* For each monkey, we pooled choice data for the same offered liquid rewards across sessions to compute the mean choice frequency. We first transformed the observed left-right choices into reward-based choice outcomes and fitted the binary reward choice outcomes using the binomial distribution (*binofit* function, MATLAB) to estimate the mean choice frequency and the 95 % confidence interval. Statistical significance of choice frequencies was determined based on two-tailed one-sample proportion tests against the null hypothesis, $p_0 = 0.5$. In addition, differences of choice frequencies between two samples were examined based on two-sided two-sample proportion test against the null hypothesis $\widehat{p_1} - \widehat{p_2} = 0$.

Choice repetition. We quantified choice repetition based on repeated-choice counts, which tracked how many consecutive choices for the same reward had been performed up to the current choice trial (**Fig. 1E, Fig. S1**). Starting from zero, repeated-choice counts accumulated when the chosen reward on the current trial matched the previously chosen reward and returned to zero otherwise. Therefore, each session had two strings of repeated-choice counts that tracked the numbers of repetitive choices for each reward. Repeated-choice counts for each reward were compared using a two-sided likelihood ratio test:

$$U = \frac{\widehat{\theta_1}}{\widehat{\theta_2}} \sim F(2n_1, 2n_2)$$

where $\widehat{\theta_1}$ and $\widehat{\theta_2}$ were the means of two exponential repeated-choice counts and the degrees of freedom were twice the sample numbers ($df_1 = 2n_1, df_2 = 2n_2$).

Nutrient-magnitude trade-off. To examine nutrient-magnitude trade-offs, we computed the intake of fat, sugar, and reward amount with respect to the maximally and minimally available intake in each session (**Fig. S1**). For each choice trial, we multiplied the reward amount and the nutrient concentration to calculate the amount of fat and sugar for both options. We then summed the larger nutrient amount between the options in each trial as the upper bound of the fat and sugar intake, respectively. Similarly, the smaller nutrient amounts were summed to derive the lower bound of nutrient intake. We then normalized the actual fat, sugar, and reward intake amount into percentage between these bounds,

$$\text{intake (\%)} = \frac{I - I_{min}}{I_{Max} - I_{min}} \times 100\ \%$$

where I is the actual intake of fat and sugar (g) or reward amount (mL); I_{Max} and I_{min} are the maximally and minimally available intake amount. Therefore, any monkey who aimed to maximize the reward amount should obtain 100 % of reward amount ($I = I_{Max}$). By contrast, because the controlled nutrient content was identical in both rewards, the intake of the controlled nutrient scaled with the percentage of reward intake. The intake of the manipulated

nutrient, however, should be 50 % for a reward maximizer because the reward magnitudes were randomized. The equally distributed choices should translate to the mean of the maximal and minimal intake, i.e. $E[I] = (I_{Max} + I_{min})/2$ or 50 % intake percentage. However, if the monkeys showed a preference for the manipulated nutrient, they would choose the high-nutrient reward even when it was offered in lower amounts, thereby reducing the intake of reward amount (< 100 %) in exchange for the preferred manipulated nutrient (> 50 %). The extent to which the intake of reward amount reduced from 100 % revealed the willingness of the monkeys to trade reward magnitudes for preferred nutrients.

Psychometric curves and subjective reward values. We estimated subjective reward values based on the psychometric curves that linked choice probabilities to the offered reward amounts (**Fig. 1F, Fig. 2A**). Specifically, as we used LFLS as the common reference to estimate the values of other rewards, we first binned the log offer ratios (LFLS/alternatives) into deciles and computed the binned choice frequencies of LFLS. We then fitted the choice frequencies at different offer ratios with a two-parameter logistic function:

$$P(left\ choice) = f(x) = \frac{1}{1 + e^{-k(x-x_0)}} \quad , x \in \mathbb{R}^+$$

where x was the log offer ratio between LFLS and the alternatives; x_0 was the inflection point of the logistic curve; k was a steepness constant of the curve, and e was the base of natural logarithm. The inflection point x_0 represented the specific ratio at which both rewards were chosen with equal probability (indifference point), i.e. $p(choice) = 0.5$, and signaled the relative exchange rate between rewards, i.e. one unit of reward was equally valued to x_0 unit(s) of LFLS. Therefore, an indifference point larger than ($x_0 > 1$) and a right-shifted psychometric curve revealed a positive preference for the alternative reward compared to LFLS, and vice versa (**Fig. 2A**, top). To compress the psychometric curves for better visualization while preserving noticeable changes of the indifference points, we log-transformed the offered ratios with respect to base 2 and reversed the transformation once we acquired the indifference point estimates. We further performed a 10-fold cross-validation to examine the reward value estimates by transforming the trial-by-trial offers into value equivalents of LFLS. Specifically, we computed the value equivalents of high-nutrient rewards (V) by multiplying the offered reward amount (RM) with the subjective reward value (SV) and predicted choices based on the differential LFLS-equivalent offers ($V_L - V_R$) (**Fig. 2B**),

$$V = RM \times SV$$

$$P(left\ choice) = f(x) = \frac{1}{1 + e^{-k(V_L-V_R)}}$$

The probability of left choices was again fitted with the two-parameter logistic function to assess how well the subjective reward values served as the exchange rates between rewards to explain the choices. The distribution of the adjusted R^2 in the sigmoid fit evaluated the out-of-sample validity of these value estimates.

Transitivity of relative values. To validate the reward values derived from the psychometric curves as subjective exchange rates between rewards, we examined the transitivity of these values by comparing direct relative values between reward pairs and their corresponding indirect relative rewards through another intermediate reward (**Fig. S2**). If reward values are suitable exchange rates between rewards, the direct values and the indirect values should be equal, as in the following derivation:

$$value(i,j) \times value(j,k) = \frac{v_j}{v_i} \times \frac{v_k}{v_j} = \frac{v_k}{v_i} = value(i,k)$$

where the indirect relative value from reward i to reward j ($value(i,j)$) and then from reward j to reward k ($value(j,k)$) was computed based on the multiplication of the value ratios between the relevant rewards. The result was identical to the direct relative value between reward i and reward $k(value(i,k))$. Thus, valid reward value estimates should fulfill this transitivity criterion to explain choices between rewards with different level of preferences. We performed this analysis across all pairs between the four factorial rewards, except for those

involving the HFHS reward because for some of the animals the reward estimates for the highly preferred HFHS reward were outside of the appropriate estimation range, due to the necessarily limited magnitude range we could offer in the task.

Logistic regression analysis

Mixed-effects multinomial logistic regression. We adopted mixed-effects multinomial logistic regression analysis (*fitglme* function, MATLAB) to model the animals' trial-by-trial choices. Specifically, we modelled the left-right choices excluding the first 50 trials in each session (during which associations between visual cues and liquid rewards were learned) and specified the categorical session number (*Session*) as the group variable to account for session-by-session (i.e. day-by-day) variations (random effects). We adopted the global model in which we estimated both the main effects and random effects of all the relevant regressors. The response variable was the dichotomous left ($LeftChosen = 1$) or right ($LeftChosen = 0$) trial-by-trial choice, collected from S_k sessions in monkey k ($S_k \in \mathbb{N}, k = 1,2,3$). Under the framework of generalized linear mixed models (GLMMs) with logit function as the link function, the logistic regression model can be specified as

$$logit(\pi_{ij}^{L}) = log\left(\frac{\pi(LeftChosen_{ij} = 1)}{\pi(LeftChosen_{ij} = 0)}\right) = \mathbf{x}_{ij}'\boldsymbol{\beta} + \mathbf{z}_{ij}'\mathbf{u}_i + \varepsilon_{ij} \quad ,\varepsilon_{ij} \sim Normal(0, \sigma^2)$$

where π_{ij}^{L} denotes the probability of choosing left in the jth trial of session i ($j = 1,2,\ldots,T_i \in \mathbb{N}; T_i =$ the total number of trials in session i); $\mathbf{x}_{ij}$ is a vector of trial-by-trial predictors depending on the models (fixed-effect regressors; see below) and $\mathbf{z}_{ij}$ is vector of trial-by-trial predictors nested in $\mathbf{x}_{ij}$, and the effects of these predictors vary across sessions (random-effect regressors). Under the assumption that the session-by-session variations of random effect regressors followed normal distribution with mean 0 and covariance matrix Ω,

$$\mathbf{u}_i \sim Normal(0, \Omega), \Omega = \begin{bmatrix} \sigma_{11}^2 & \cdots & 0 \\ \vdots & \ddots & \vdots \\ \sigma_{1i}^2 & \cdots & \sigma_{ii}^2 \end{bmatrix}, i = 1,2,\ldots,S_k \in \mathbb{N}, k = 1,2,3$$

the model estimated the coefficients of fixed-effect regressors, $\boldsymbol{\beta}$, and the session-wise variations of the random-effect regressors, $\mathbf{u}_i$. The estimated left-right choice responses, p_{ij}^{L}, were derived by reverse logit function conditional on the session-wise random effects ($\mathbf{u}_i$), and the session-wise regression coefficients ($\boldsymbol{\eta}_i$) were derived from the fixed-effect coefficients ($\boldsymbol{\beta}$) and the session-wise calibration terms ($\mathbf{u}_i$).

$$p_{ij}^{L} = P(LeftChosen = 1|\mathbf{u}_i) = \frac{\exp(\mathbf{x}_{ij}'\boldsymbol{\beta} + \mathbf{z}_{ij}'\mathbf{u}_i)}{1 + \exp(\mathbf{x}_{ij}'\boldsymbol{\beta} + \mathbf{z}_{ij}'\mathbf{u}_i)} \in [0,1], \qquad \boldsymbol{\eta}_i = \boldsymbol{\beta} + \mathbf{u}_i$$

Regression models.

1. **Nutrient model.** In the main nutrient model (**Table S2**), we modeled basic nutrient sensitivities while controlling task-related regressors including the position of the liquid-delivery spout (*Spout*) and the presentation order of visual cues (*LeftFirst*) in a mixed-effect model. Importantly, we specified the categorical session number (*Session*) as the group variable to address session-wise variations of nutrient sensitivities as follows,

$$\boldsymbol{logit(LeftChosen) = \beta_0 + \beta_1 \times LeftFirst + \beta_2 \times Spout + \beta_3 \times RM}$$
$$\boldsymbol{+\beta_4 \times SugarLv + \beta_5 \times FatLv + \beta_6 \times SugarLv \times FatLv \mid Session}$$

where $LeftFirst$ indicated whether the left option was shown first (1, if the left option was shown first; 0 if the right option was shown first), $Spout$ indicated the spout channel that delivered the left reward option (1, if left, 0 if right). RM represented the left offer magnitudes minus the right offer magnitudes, whereas $FatLv$ and $SugarLv$ coded the ordinal left-right nutrient level differences (1, if left > right; 0, if left = right; -1, if left < right)

and $SugarLv \times FatLv$ captured the additional fat-sugar interactions (1, if the left option was both high-fat and high-sugar, but the right was not; -1, if the opposite, and 0, if otherwise).

2. **Energy model.** To test the hypothesis of energy maximization, we combined reward magnitudes and nutrient content into a single energy-content regressor and included the energy difference between left-right options to construct the energy model as follows (**Table S2**),

$$logit(LeftChosen) = \beta_0 + \beta_1 \times LeftFirst + \beta_2 \times CS1Left + \beta_3 RM + \beta_4 \times Energy \mid Session$$

   where the newly included $Energy$ regressor represented the left-right differences in actual energy content (kcal).

3. **Nutrient history model.** We explored how past fat and sugar choices influenced current nutrient sensitivities by including interaction terms between current nutrient sensitivities and within-nutrient (sugar→sugar, fat→fat) or across-nutrient (sugar→fat, fat→sugar) feedback up to 10 trials prior to current trial (**Table S2**).

$$\begin{aligned} logit(LeftChosen) = (\ &\beta_0 + \beta_1 \times LeftFirst + \beta_2 \times CS1Left + \beta_3 \times RM \\ &+ \beta_4 \times SugarLV + \beta_5 \times FatLv \mid Session) \\ &+ \textstyle\sum_{k=1}^{10}(\beta_{5+k} \times SugarLv \times SugarIntake_k) \\ &+ \textstyle\sum_{k=1}^{10}(\beta_{15+k} \times FatLv \times FatIntake_k) \\ &+ \textstyle\sum_{k=1}^{10}(\beta_{25+k} \times SugarLv \times FatIntake_k) \\ &+ \textstyle\sum_{k=1}^{10}(\beta_{35+k} \times FatLv \times SugarIntake_k) \end{aligned}$$

   In this model, in addition to the nutrient model, we computed the intake of fat and sugar up to 10 trials prior to the current trial ($FatIntake_k$, $SugarIntake_k$, $k = 1,2,3,\dots,10$). The within-nutrient effects were modeled by the interactions between past sugar intake and current sugar sensitivity, as well as past fat intake and current fat sensitivity; the across-nutrient effects were captured by the influences of past sugar intake on current fat sensitivity and the influences of past fat intake on current sugar sensitivity.

Model comparison. We compared the performance of regression models (**Fig. 4E**) based on the Akaike Information Criteria (AIC),

$$AIC = 2k - 2logL$$

where k is the number of parameters in the model and L is the maximal likelihood of the model predictions given the actual data. Because the relative likelihood of model t (RL_t) suggested by the AIC difference is

$$RL_t = \exp\left(\frac{AIC_0 - AIC_t}{2}\right)$$

where AIC_0 and AIC_t are the AIC values of the reference model and model t, respectively, we accepted that a model was better if the model likelihood was 5 times more than the competing models, i.e. $\Delta AIC = AIC_0 - AIC_t > 2log5 \approx 3.22$. We constructed the main nutrient model by including significant task-related regressors based on AIC criteria for all three monkeys. We then performed similar model comparison between the nutrient model and the energy model to test the energy maximization hypothesis against the nutrient valuation strategy (**Fig. 4E**).

Cross-prediction validation. We examined the robustness of the models across sessions, across flavors (**Fig. 2E**), and across animals (**Fig. 2F, Fig. S5**). The cross-prediction analyses validated the regression models outside of the training samples. Specifically, we first separated the data into mutually exclusive training and testing sets. We then predicted choices in the testing set based on the regression coefficients derived from the training data. The performance of the cross-prediction was evaluated by (McFadden's) cross-validated pseudo-R^2,

$$\text{Cross-validated pseudo-R}^2 = 1 - \frac{LL_{nutrient}}{LL_{null}}$$

which was based on the log-likelihood ratio of choices predicted by the nutrient model ($LL_{nutrient}$) and the intercept model (LL_{null}). Higher cross-validated pseudo-R^2 indicated better cross prediction performance, therefore more robust nutrient-value functions across conditions. Based on this concept, in the cross-session prediction, we sequentially left out one session as testing set and fitted the nutrient model in the remaining training data. We then reported the mean cross-validated pseudo-R^2 to indicate the stability of the nutrient model across testing sessions. In the cross-flavor prediction (**Fig. 2E**), we took turns using choice data in one flavor as training set to predict choices in another flavor. The cross-predicted pseudo-R^2 was reported in the confusion matrix. Lastly, in the cross-animal prediction (**Fig. 2F, Fig. S5**), we used the nutrient-value function from one monkey to predict the other two monkeys' choices. We first randomly selected one flavor-matched testing session from each of the three monkeys. We then fitted the nutrient model in one of the monkeys (training monkey) using choices excluding the left-out session and predicted choices in the three testing sessions. We compared the prediction performance on the other two monkeys (testing monkeys) to that on the training monkey. Importantly, we truncated the testing sessions to identical trial numbers to ensure comparability.

Preference dissimilarity index (PDI). To quantify the distinctiveness of choice patterns across monkeys, we defined the preference dissimilarity index (PDI) as the bidirectional average ratio of the log-likelihood (LL)

$$PDI_{mn} = \log\left[\frac{1}{2}\left(\frac{LL_{mn}}{LL_{mm}} + \frac{LL_{nm}}{LL_{nn}}\right)\right]$$

where LL_{mn} denotes the log-likelihoods of using trained nutrient preference of monkey m to predict choices of monkey n, and LL_{nm} the opposite; LL_{mm} and LL_{nn} were self-predicted reference models based on the monkey's own nutrient preferences. The PDI compared the cross-predictability to self-predictability, with PDI = 1 indicating comparable cross-predictability based on self and other nutrient preferences. PDIs larger than 1 suggested inconsistency between other-predicted and the reference self-predicted choices (distinct choice patterns). The cross-prediction was repeated for 1,000 iterations between each pair of the monkeys to compute the average log likelihood ratios for pairwise PDIs. The results were visualized in a preference triangle using pairwise PDIs as side-lengths; therefore, longer between-animal distances indicated more distinct choice patterns of the two connected animals (**Fig. 2F Fig. S5**). The importance of fat and sugar in explaining the individual differences of choice patterns were estimated by repeating the cross-animal predictions after systematically including fat and sugar regressors in the nutrient model. The nutrient contribution was estimated by the percentage change of PDI with and without the nutrient regressor in the nutrient model.

$$\text{Nutrient contribution} = \frac{PDI_{nutrient} - PDI_{null}}{PDI_{full} - PDI_{null}}$$

With the null model containing only the reward magnitudes and task-related control variables, the nutrient contribution of choice discrepancies was quantified as the increase in the PDI after adding specific nutrient regressor into the null model ($PDI_{nutrient}$) normalized with the whole range of PDI, which was bounded by the full nutrient model (PDI_{full}) and the null model (PDI_{null}) (**Fig. 2G**).

Mediation analysis. We adopted mediation analysis (9, 10) in logistic regressions to assess possible causal relationships between the nutrient content, oral texture parameters and reward choices. The framework of mediation analysis involved three components (**Fig. 3D**): *Component 1 (path c)* — fat and sugar contents were significant predictors for choices (total effect); *Component 2 (path a)* — fat and sugar content (predictors) were correlated with the texture parameters (mediators); *Component 3 (path c')* — after including the mediators into

the regression model, they replaced the effects of the original predictors, either completely (complete mediation) or partially (partial mediation). The direct effect (*path c'*) of the nutrient content (predictors) on choices (outcome) was defined as the coefficients of nutrient content (predictors) after controlling the texture parameters (mediators). The mediation effect (indirect effect = c-c') was then quantified as the coefficient differences between the total effect and the direct effect.

Specifically, we examined whether the oral texture parameters replaced the effects of fat and sugar in the nutrient model by independently including viscosity and CSF into the nutrient model (**Table S2**):

$$logit(LeftChosen) = \beta_0 + \beta_1 \times LeftFirst + \beta_2 \times Spout + \beta_3 \times RM + \beta_4 \times SugarLV + \beta_5 \times FatLv + \beta_6 \times SugarLV \times FatLv + \beta_7 \times Viscosity \mid Session$$

$$logit(LeftChosen) = \beta_0 + \beta_1 \times LeftFirst + \beta_2 \times Spout + \beta_3 \times RM + \beta_4 \times SugarLV + \beta_5 \times FatLv + \beta_6 \times SugarLV \times FatLv + \beta_7 \times CSF \mid Session$$

The differences of fat and sugar regression coefficients before (nutrient model, total effect: c) and after including texture parameters (texture model, direct effect: c') in all three monkeys showed that viscosity and CSF partially replaced both fat and sugar sensitivities (**Fig. 3E**). We tested the significance of the mediation effects using a bootstrap analysis (11, 12) with 1,000 iterations. We accepted significant mediation effects if the iterated distributions of the mediation effects significantly deviated from zero.

Structural Equation Modelling. Based on the framework of structural equation modelling (SEM), we combined three logistic regressions in the path analysis to describe the relationships between nutrient content, food texture parameters and choices (**Fig. 3F**). The first two regressions recapitulated how fat and sugar content influenced viscosity and sliding friction:

$$Viscosity = \beta_0 + \beta_1 \times FatLv + \beta_2 \times SugarLV$$
$$CSF = \beta_0 + \beta_1 \times FatLv + \beta_2 \times SugarLV$$

The third regression characterized the influences of food textures and the direct influences of sugar content independent of its texture (direct effect) on choices:

$$\text{Total effect:} \quad \text{logit}(LeftChosen) = \beta_0 + \beta_1 \times SugarLv + \beta_2 \times FatLv$$
$$\text{Direct effect:} \quad \text{logit}(LeftChosen) = \beta'_0 + \beta'_1 \times SugarLV + \beta'_2 \times Viscosity + \beta'_3 \times CSF$$

The differences between the regression coefficients for sugar level in the two models ($\beta_1 - \beta'_1$) were defined as the mediation effect of the texture parameters between sugar content and choices. We performed a bootstrap test with 1,000 iterations to evaluate the significance of these regression coefficients and the mediation effects. All path coefficients were normalized and expressed in the path diagrams to indicate how fat and sugar content could change the food textures to influence reward choices.

Reward space choice trajectories. In the reward space, starting from the origin, we plotted the cumulative choices between the two options against each other (**Fig. 4A**, **B**). We normalized the choice counts to the total trial number in each session and averaged the cumulative choices across sessions. Thus, indiscriminate choices would show a choice trajectory that follows the 45-degree unity line and an endpoint that rests on the midpoint of the hypotenuse. Conversely, deviations away from the unity line suggest a choice bias and the continuous trajectory describes the changing choice patterns within the session. In addition, we compared reference trajectories based on three strategies that maximized energy, sugar, or fat, respectively. Specifically, we first computed the target component (energy, fat, or sugar) of both options in each trial. The simulated maximizers then chose on each trial the option with the higher target component and chose randomly when the two options matched in target component.

Nutrient space choice trajectories. In the nutrient space, we converted the choice trajectories from the reward space to visualize the changing patterns of nutrient sensitivity within sessions (**Fig. 4C**, **D**). Specifically, we first computed the energy intake from fat and sugar (kcal) on each trial, based on the chosen reward magnitude and the nutrient composition of the chosen reward. Next, we normalized the trial-by-trial nutrient-specific energy intake to the total trial number in each session before averaging them to derive the final trajectories. For visualization, we plotted the smoothed energy intake from sugar against fat (kcal/kcal) across sessions. Thus, in the isocaloric comparison, the slope of the trajectory revealed the animals' trade-off between fat and sugar as source of energy, indicated by the angle θ between the trajectory and the horizontal axis,

$$\theta = tan^{-1}\left(\frac{\text{Energy intake from sugar }(kcal)}{\text{Energy intake from fat }(kcal)}\right)$$

Likewise, the three reference trajectories in the reward space were transformed into the nutrient space, to indicate the relative nutrient intake in the three maximizing strategies.

Geometric Framework for Nutrition (GFN). We used the right-angled mixer triangle developed by Raubenheimer which implements a proportion-based variant of the Geometric Framework for Nutrition (GFN) (13, 14) in which the available food compositions, reference nutritional targets, and the actual nutrient-intake balance could be analyzed in a common framework (**Fig. 5A**). The compositions of food rewards were plotted in a mixer triangle (13) based on the percentage contribution of fat and sugar to total energy content. The actual nutrient-intake balance ($\mathbf{N}^*$, a vector of nutrient composition in percentage of total energy) was calculated based on the ratio of consumed fat and sugar amount from the choices:

$$\mathbf{N}^* = \frac{\text{Total energy intake from nutrients (kcal)}}{\text{Total energy intake (kcal)}} \times 100\% = \frac{I_N}{I_{total}} \times 100\%$$

Because nutrients can be acquired through reward A or reward B, the total intake can be separated based on the source of rewards,

$$I_N = E_A \cdot \mathbf{N}_A \cdot \underbrace{\textstyle\sum_{i=1}^{n} RM_i^A \cdot I_i^A}_{\text{intake amount of reward A}} + E_B \cdot \mathbf{N}_B \cdot \underbrace{\textstyle\sum_{i=1}^{n} RM_i^B \cdot I_i^B}_{\text{intake amount of reward B}}$$

$$I_{total} = E_A \cdot \underbrace{\textstyle\sum_{i=1}^{n} RM_i^A \cdot I_i^A}_{\text{intake amount of reward A}} + E_B \cdot \underbrace{\textstyle\sum_{i=1}^{n} RM_i^B \cdot I_i^B}_{\text{intake amount of reward B}}$$

where RM_i^A and RM_i^B were the offered magnitudes (mL) of reward A and B on trial i across total n trials; I^A and I^B were indicator functions whose values were 1 only when the specific reward was chosen, and 0 if otherwise. The intake amounts of rewards were then multiplied by the energy density, E_A and E_B (kcal/mL), and the nutrient composition, N_A and N_B (% energy), to compute the energy intake from specific nutrients. Because the reward compositions ($\mathbf{N_A}$, $\mathbf{N}_B$) were constant within sessions, the geometric representations of final nutrient balance were interpolations between the two points for reward options, $\mathbf{N}_A$ and $\mathbf{N}_B$, weighted by the energy intake contributed by each reward.

$$\mathbf{N}^* = \frac{I_A}{I_A + I_B} \cdot \mathbf{N}_A + \frac{I_B}{I_A + I_B} \cdot \mathbf{N}_B = \alpha \cdot \mathbf{N}_A + \beta \cdot \mathbf{N}_B \in \overline{\mathbf{N}_A\mathbf{N}_B}$$
$$,\alpha = \frac{I_A}{I_A + I_B} \in [0,1], \qquad \beta = \frac{I_B}{I_A + I_B} \in [0,1], \qquad \alpha + \beta = 1$$

Nutrient reference comparison. We compared the nutrient intake balance derived from the animals' choices with two nutrient reference points, a recommended ('optimal') diet composition for adult macaques (15) and macaque milk (16). Because the actual nutrient balance should lie on the segments that connected the reward options, we orthogonally projected the reference points onto the lines that connected LFLS and HFLS or LFHS, using vector orthogonal projection as below (**Fig. 5C, Fig. 5D**):

$$\overrightarrow{AR'} = R' - A = \frac{\overrightarrow{AR} \cdot \overrightarrow{AB}}{\left\|\overrightarrow{AB}\right\|^2} \cdot \overrightarrow{AB}$$

where A and B were nutrient compositions of reward A and reward B, R was the nutrient reference point and R' was its projection onto line $\overleftrightarrow{AB}$. These projection points were the closest achievable targets that served as surrogate nutrient references in these comparisons.

Reinforcement Learning (RL) simulation

Reversal-learning task. We simulated a reversal-learning task involving binary choices between high-nutrient (H) and low-nutrient (L) reward options. Each option was associated with a specific reward probability, in this case $P(H) = 0.6, P(L) = 0.4$ (**Fig. S9A**). The reward probability was reversed regularly without notification every 50 trials, e.g. $P(H) = 0.6 \longrightarrow 0.4, P(L) = 0.4 \longrightarrow 0.6$. Therefore, the agent should track the changing reward values through trial and error. This basic reversal-learning task has been widely used as a paradigm for adaptive learning in neuroscience and has been successfully modelled by RL models (17-19).

Standard RL model. In the standard RL model, we adopted the basic form of a Q-learning algorithm that followed the Rescorla-Wagner learning rule (20, 21) and performed 100 repetitions of choice simulations in the reversal learning task (**Fig. 6A**). The agent tracked the values of the high-nutrient (H) and low-nutrient (L) rewards through trial-and-error (Q^H: value of the high-nutrient reward; Q^L: value of the low-nutrient reward) and made choices based on the value difference (δ) in each trial. Specifically, starting with equal values for both rewards and indiscriminate choices ($Q_1^H = Q_1^L = 0.5$; $P(H)_1 = P(L)_1 = 0.5$), the agent computed in each choice trial the value difference based on the latest value information (δ_{t-1}) and transformed it via the softmax function into the choice probability,

$$\delta_{t-1} = Q_{t-1}^H - Q_{t-1}^L$$
$$\pi(H)_t = \frac{1}{1 + \exp(-\delta_{t-1})} \in [0,1]$$

where $\pi(H)_t$ denoted the probability of choosing the high-nutrient reward on trial t, and the low-nutrient choice probability was $\pi(L)_t = 1 - \pi(H)_t$. We then dichotomized the choice probability at 0.5 to for the choice actions,

$$A(H)_t = \begin{cases} 1 & , \text{if } \pi(H)_t > 0.5 \\ Y & , \text{if } \pi(H)_t = 0.5 \\ 0 & , \text{if } \pi(H)_t < 0.5 \end{cases} \qquad A(L)_t = \begin{cases} 1 & , \text{if } A(H)_t = 0 \\ 0 & , \text{if } A(H)_t = 1. \end{cases} \qquad , Y \sim B(1,0.5)$$

The choice action for the high-nutrient reward $A(H)_t$ was 1 when the choice probability was larger than 0.5, and was 0 if otherwise. In case of equal choice probability ($\pi(H)_t = \pi(L)_t = 0.5$), the agent flipped a fair coin (Bernoulli trial) to decide which reward to choose. The subsequent reward outcomes of the choices were randomly drawn depending on the task-assigned reward probability, which alternated every 50 trials in the reversal-learning task. The received rewarded value was 1 if the agent received the reward and was 0 if otherwise.

$$R_t^H = R_t^L = \begin{cases} 1 & , \text{if rewarded} \\ 0 & , \text{if unrewarded} \end{cases}$$

Importantly, in the standard RL model, the values for both rewards were identical, irrespective of their nutrient composition. This value specification was later modified in the nutrient RL model to incorporate the nutrient preferences into the RL framework.

Nutrient-sensitive RL model. In the nutrient-sensitive model, we extended the standard RL model by assigning higher reward outcomes for the high-nutrient reward than for the low-

nutrient reward, when rewarded. The higher value for the high-nutrient reward was controlled by a nutrient-sensitivity parameter $\eta \in [0,1)$ as follows (**Fig. 6B, Fig. S9**),

$$R_t^H = \begin{cases} \frac{1}{1-\eta} \geq 1 & \text{,if rewarded} \\ 0 & \text{, if unrewarded} \end{cases} \quad R_t^L = \begin{cases} 1 & \text{, if rewarded} \\ 0 & \text{, if unrewarded} \end{cases}$$

The single nutrient-sensitivity parameter η, created a continuous spectrum of nutrient-sensitive RL models, which degenerated to the standard RL model when $\eta = 0$ and converged into high-nutrient only choices regardless of the reward probability when $\eta \to 1$ ($R_t^H \to \infty$). By contrast, the reward outcome for the low-nutrient reward remained unchanged as the standard RL model.

Economic choice theory simulation

Nutrient indifference map. We simulated each monkey's choices in the nutrient choice task in which we systematically compared randomized amounts of LFLS with rewards across combinations of interpolated fat and sugar content (**Fig. S10**). We systematically sampled LFLS ($FatLv = 0$, $SugarLv = 0$) against rewards with combinations of fat and sugar level from 0.1 to 1 with in steps of 0.1. The 10,000 simulated choices were based on the regression coefficients derived from each monkey (**Fig. 2C**). We then based on the simulated choices to estimate the reward values for each fat-sugar combination using the indifference points on the psychometric curves. In the model, we log-transformed the fat and sugar level to follow the formulation of Cobb-Douglas utility function (22, 23), which created non-overlapping, non-decreasing, and negative-sloped indifference curves that have been widely used in economic studies.

$$logit(\pi_{ij}^L) = k_{ij} + \alpha_i \cdot log\left(\frac{FatLv_L}{FatLv_R}\right) + \beta_i \cdot log\left(\frac{SugarLv_L}{SugarLv_R}\right) + \varepsilon_{ij} \ , \varepsilon_{ij} \sim Normal(0, \sigma^2)$$

where π_{ij}^L denotes the probability of choosing left in the jth trial in session i, k_{ij} represents the aggregated task-related parameters as in the nutrient model, ε_{ij} is the Gaussian random error.

Extension of indifference analysis. We proposed an approach for indifference analysis between goods with common ingredients but different compositions (**Fig. 6D**). Specifically, we constructed four example composite food rewards, each with different compositions of fat and sugar (fat/sugar): A (70%/30%), B (10%/90%), C (90%/10%), D (20%/80%). We assumed that the values for fat and sugar followed the exponential utility function,

$$v(c) = \begin{cases} (1 - e^{-ac})/a & a \neq 0 \\ c & a = 0 \end{cases}$$

where c is the quantity of the nutrients and a is a risk attitude parameter that determines the curvature of the function. In addition, we also assumed that the values for fat and sugar were additive; therefore, the value of reward *X* was the weighted sum of values of its nutrient constituents,

$$U(X) = U(f, s) = \frac{1 - exp(-a_f \cdot f)}{a_f} \cdot w_f + \frac{1 - exp(-a_s \cdot s)}{a_s} \cdot w_s$$

Without loss of generality, we set the risk attitude parameters for fat and sugar as $a_f = 2$, $a_s = 5$, and the weights that integrated values of fat and sugar into the composite reward values as $w_f = 0.3$, $w_s = 0.7$. The slightly larger parameters for sugar reflected the observed stronger influences of sugar content than fat content on choices. Next, we illustrated how four reward bundles (a, b, c, d) and their relative preference ranking linearly transformed from reward space A-B to the nutrient space, and finally to reward space C-D. Specifically, each bundle point P_k ($k = 1,2,3,4$) in the reward space A-B was linearly transformed into the nutrient space,

$$P'_k = T_N P_k = N_{AB} P_k = \begin{bmatrix} N_f^A & N_s^A \\ N_f^B & N_s^B \end{bmatrix} \begin{bmatrix} P_k^f \\ P_k^s \end{bmatrix}$$

where P'_k was the transformed bundle point in the nutrient space, the transformation matrix to nutrient space (T_N) was the nutrient composition matrix (N_{AB}) that included the nutrient vectors of the composite reward A and B (N_f^A: fat content in reward A, N_s^A: sugar content in reward A; N_f^B: fat content in reward B, N_s^B: sugar content in reward B). Similarly, the same four bundle points can be again linearly transformed into reward space C-D, which was defined by rewards with the same ingredients as reward A,B but with different compositions.

$$P''_k = T_R P'_k = {N_{CD}}^{-1} P'_k = \begin{bmatrix} N_f^C & N_s^C \\ N_f^D & N_s^D \end{bmatrix}^{-1} \begin{bmatrix} P'^f_k \\ P'^s_k \end{bmatrix}$$

Each bundle point in the reward space C-D P''_k was transformed from those in the nutrient space P'_k by multiplying the points with the transformation matrix for reward space (T_R), which was the inverse of the nutrient composition matrix that included the nutrient vectors of reward C and D (N_{CD}). Notably, the relative rankings between the four bundles were preserved throughout the transformation. This value-preserving property illustrated that the same choice analysis could be performed in the nutrient space or in reward spaces constructed by rewards with different compositions, therefore providing a unifying framework that links indifference analyses across different reward sets via their common ingredients.

Human psychophysical experiment. Healthy, non-obese participants (N = 23, aged 18-21, 15 male) gave written informed consent and participated in an experiment that involved sampling and psychophysically evaluating liquid rewards. The experiment was approved by the Local Research Ethics Committee of the Cambridgeshire Health Authority. The rewards were the same stimuli as used in our monkey experiment, but prepared specifically for human testing, and included the four factorial rewards, a cream-based stimulus, water and water-diluted fruit juice concentrate (an additional stimulus involving a food-thickener was tested in a subset of 14 subjects). All stimuli were blackcurrant-flavored. Subjects sampled and swallowed 1.5 mL of each stimulus from opaque cups in randomized order; after sampling a stimulus, subjects gave psychophysical ratings on a touchpad and rinsed their mouth with water before sampling the next stimulus. Each stimulus was sampled six times. Rating scales ranged from 1 to 10, with endpoints labelled as ‘none’ and ‘very strong’. Ratings were z-scored before analysis.

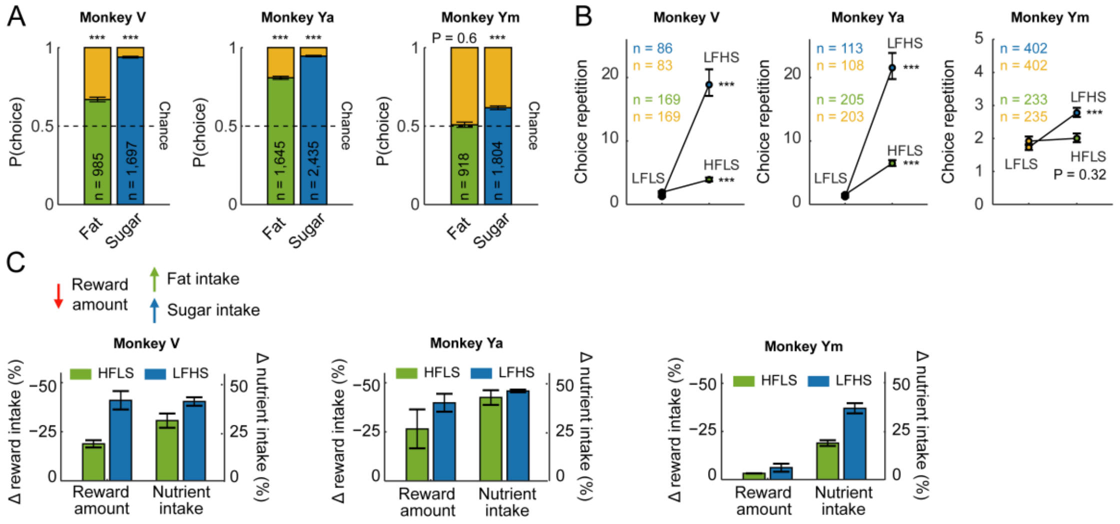

Fig S1. Preferences for fat and sugar rewards across monkeys. Choice frequencies, choice repetitions and nutrient-magnitude trade-offs suggested preferences for high-nutrient rewards. **(A)** Choice frequencies (± s.e.m.) for high-fat (HFLS) and high-sugar (LFHS) rewards when compared with the low-nutrient (LFLS) reference option in the three animals (***: $P < 1.0 \times 10^{-10}$, binomial test). **(B)** Repeated-choice counts. All three monkeys chose high-fat and high-sugar rewards more repeatedly (all $P < 0.001$), except high-fat reward for monkey Ym, likelihood ratio test.; error bars: s.e.m.) **(C)** Trade-offs between nutrient content and reward amounts. The monkeys' choices reduced total intake of reward amount (left bars, % of total offered amount in each session) and increased intake of fat and sugar (right bars, % of total offered nutrients in each session; error bars: s.e.m.).

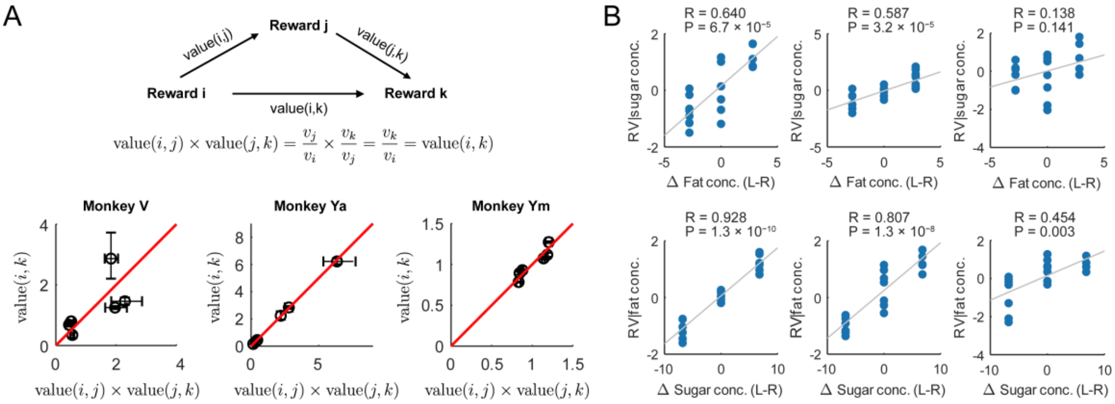

Fig S2. Transitive reward values reflected nutrient composition. (A) Reward values are transitive if preferences are preserved even after intermediate comparisons between rewards. We validated transitivity of the reward values by comparing the direct and indirect value estimates between rewards. The scatter plots (± s.e.m.) show that direct relative values between reward i and k, $\text{value}(i,k)$, are identical to the indirect values via another reward j, $\text{value}(i,j) \times \text{value}(j,k)$, in all three monkeys (see *Methods*). **(B)** The reward value estimates are significantly correlated with the fat and sugar content of the offered rewards in all three monkeys, except the effect of fat content in monkey Ym.

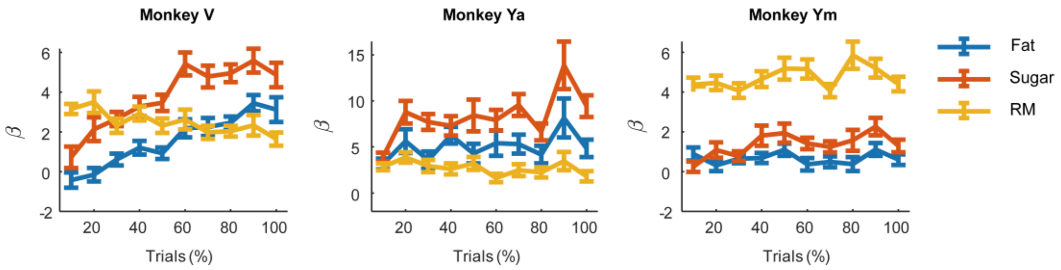

Fig S3. Stability of fat and sugar regression coefficients within testing sessions. Regression coefficients (± s.e.m.) for fat, sugar and reward magnitude (RM) from the nutrient model fitted to deciles of consecutive trial-windows in each testing session. Following initial learning of associations between conditioned stimuli and rewards coefficients for fat (blue) and sugar (green) were typically stable or increased throughout the session, compared to constant or slightly decreased coefficients for magnitude (yellow).

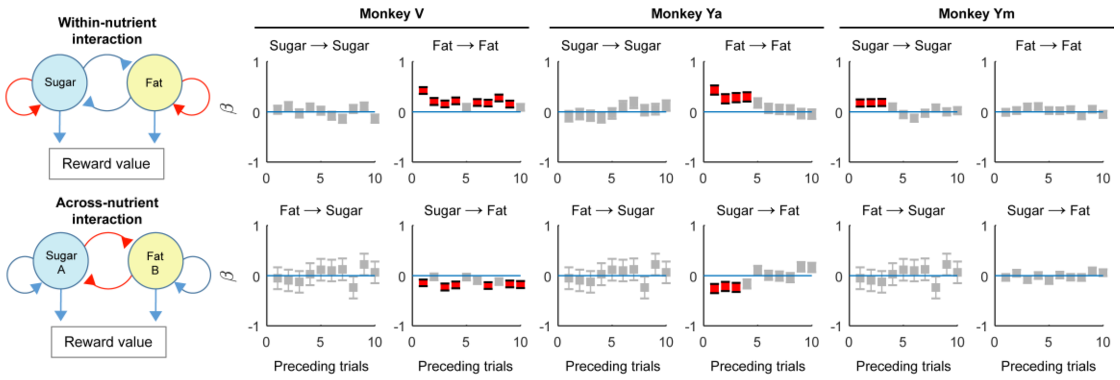

Fig S4. Nutrient-history effects on choices. Regression coefficients (± s.e.m.) from the 'nutrient history model' (see *Methods*) that extended our main nutrient model with coefficients for nutrient history, obtained by pooling trials across sessions within each animal. In monkey V and Ya, recent fat choices facilitated current-trial fat choice; whereas recent sugar choices inhibited current-trial fat choice (upper right panel, significant positive fat→fat weights and lower right panel, negative sugar→fat weights). These effects were absent in monkey Ym. However, monkey Ym showed a positive sugar feedback on sugar choices, which was not seen in the other two animals.

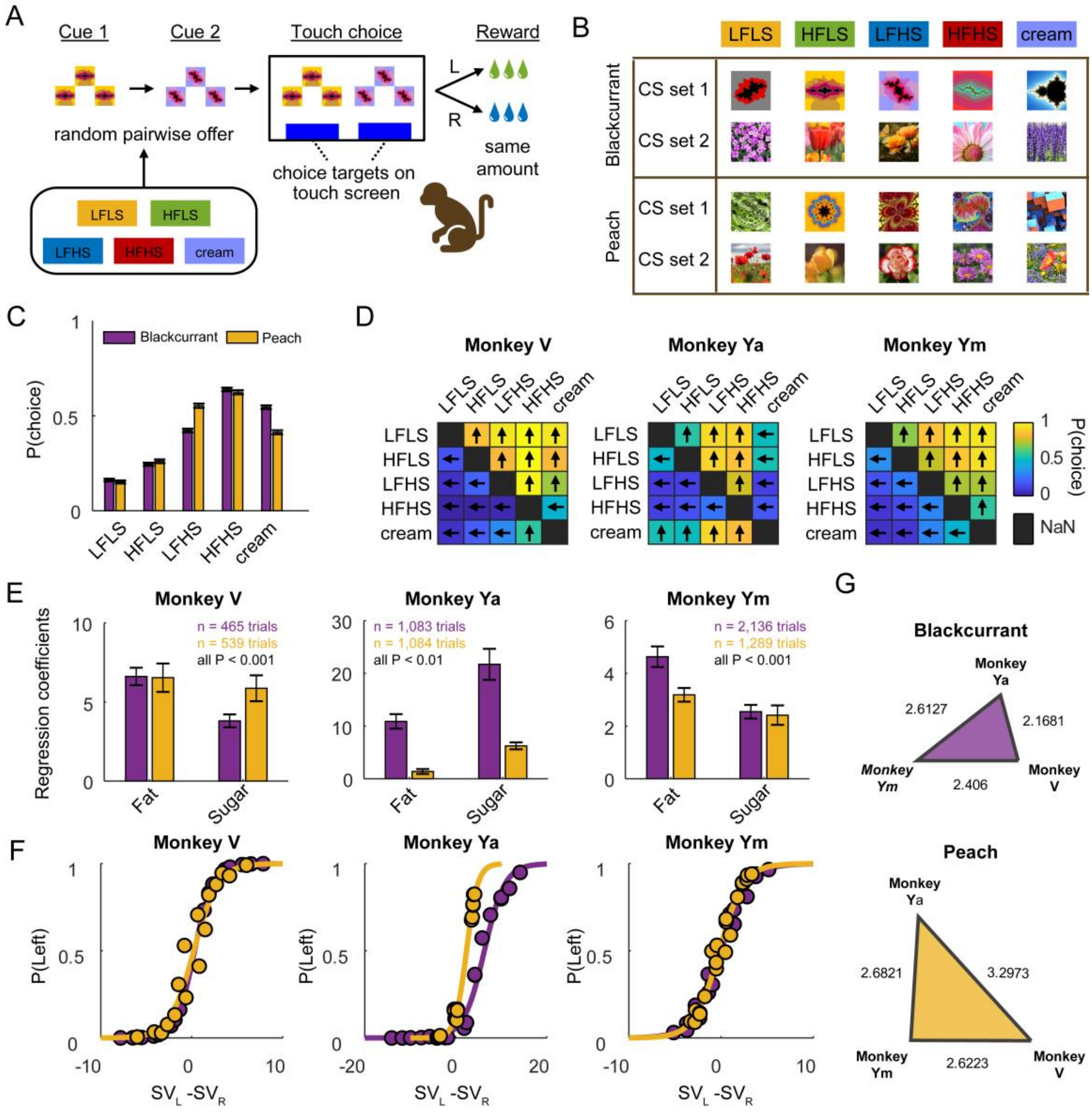

Fig S5. Results in the alternative choice task. (A) Task design. Monkeys chose between sequentially presented choice options offered in constant amounts. Options were drawn from a set of five reward stimuli, including the four factorial nutrient-rewards and a cream-based high-fat low-sugar reward (‘cream’). **(B)** Rewards were cued by two sets of pre-trained conditioned stimuli; flavors were matched (blackcurrant or peach) within the same session. **(C)** Choice frequencies for the tested rewards across sessions and animals, shown separately for the two flavors. **(D)** Pairwise comparisons between the tested rewards. Color scale represents choice frequency; arrow direction indicates the preferred reward. **(E)** Regression coefficients of fat and sugar content from mixed-effects logistic regressions fitted separately for the two flavors. **(F)** Psychometric functions show that choice frequency followed the difference in subjective value derived from the regression model (E). **(G)** Geometry of cross-animal predictions visualized choice discrepancies as triangles, with preference dissimilarity indices (PDI, see Method) as side lengths (stated as numbers on the sides).

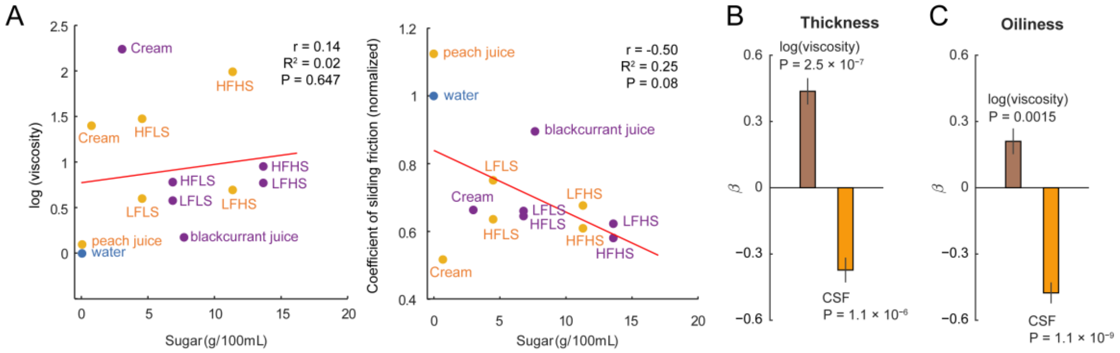

Fig S6. Relationship between sugar and food-texture parameters; human psychophysical experiment. (A) Viscosity (left) and CSF (right) as a function of sugar content in our stimulus set for the two flavors (orange: peach; purple: blackcurrant) (linear regressions). **(B, C)** Human psychophysical experiment: Relationships between texture parameters and subjective thickness and oiliness ratings, based on the same stimulus set as in the monkey experiments (blackcurrant flavor). **(B)** Texture influences on thickness ratings. Standardized regression coefficients (mean across subjects ± s.e.m.) from a multiple regression model with regressors including log(viscosity) and CSF, and thickness ratings as dependent variable. The regression was significant in 23/23 subjects with mean R^2 across subjects of 0.589 ± 0.032 (s.e.m.). Effect sizes of viscosity and CSF were not significantly different (P = 0.549, t-test on unsigned regression coefficients). **(C)** Texture influences on oiliness ratings. Standardized regression coefficients (mean across subjects ± s.e.m.) from a multiple regression model with regressors including log(viscosity) and CSF, and oiliness ratings as dependent variable. The regression was significant in 22/23 subjects with mean R^2 across subjects of 0.473 ± 0.041 (s.e.m.). The (unsigned) regression coefficient for CSF on oiliness was significantly larger than that for viscosity (P = 0.011, t-test on unsigned regression coefficients).

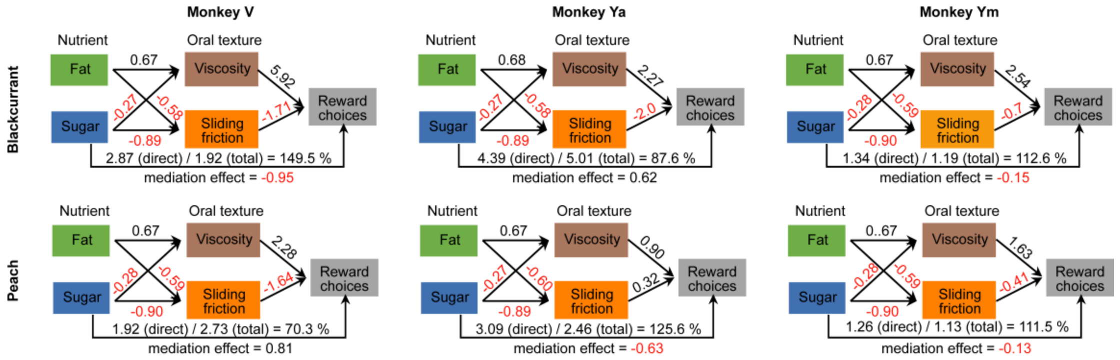

Fig S7. Path analysis between nutrients, food textures and choices for the alternative choice task. Path analysis based on the framework of structural equation modelling (SEM) and path coefficients. The effect of fat was completely mediated by the food textures, and thus removed from the path diagram. However, sugar had a direct effect on choices independent of food textures. Significance of coefficients was derived from bootstrap (1,000 iterations).

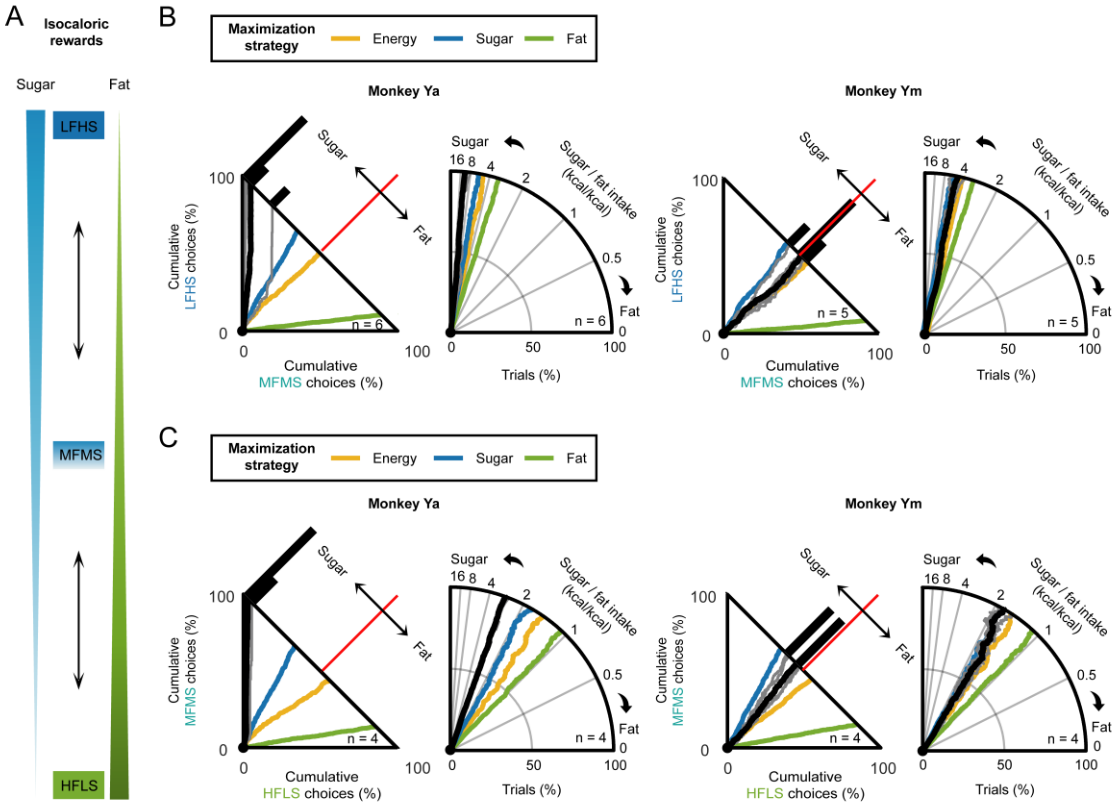

Fig S8. Choice trajectories in reward and nutrient space for choices involving medium-fat and medium-sugar rewards. (A) Schematic of tests: Monkeys chose between a medium-fat medium-sugar reward (MFMS) and isocaloric low-fat high-sugar (LFHS) or high-fat low-sugar (HFLS) rewards. **(B)** Cumulative choices for the two tested animals (black: mean trajectory of actual choices, grey: single-session trajectories; colors: simulated choice trajectories based on reference strategies that maximized calories, fat, or sugar). **(C)** Choice trajectories transformed from reward space into nutrient space (same sessions as in (C); black: actual choices; colors: reference simulated choices).

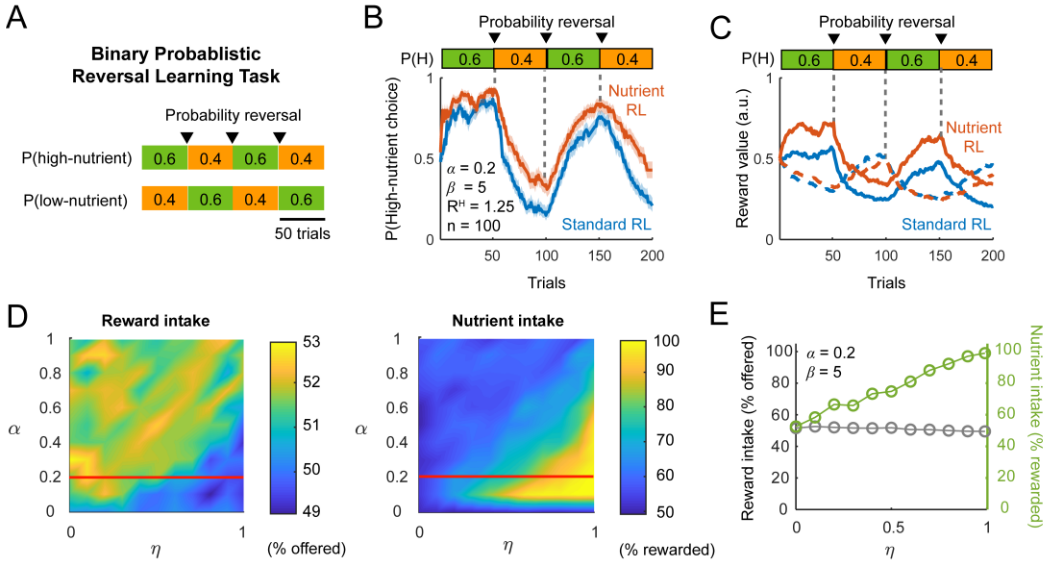

Fig S9. Nutrient-sensitive reinforcement learning simulation results. (A) Schematic of simulated binary choice probabilistic reversal-learning task. The probability of receiving high- or low-nutrient reward reversed every 50 trials. **(B)** Frequency of high-nutrient choices in the nutrient-sensitive reinforcement-learning model (red) and a standard reinforcement-learning model (blue) shown across probability reversals. **(C)** Reward values for the high-nutrient option (solid lines) and low-nutrient option (dashed lines) shown for both models across probability reversals. **(D)** Reward and nutrient outcomes across learning rates and nutrient-sensitivity parameters. Left: Color map represents obtained reward outcomes (% of offered rewards). Right: Color map represents obtained nutrient outcomes (% of rewarded trials). Results in the paper are shown for $\alpha = 0.2$ and $\eta = 0.2$; inverse temperature parameter β was fixed at 5. **(E)** Reward and nutrient intake as a function of the nutrient-sensitivity parameter. Reward intake was not affected by the nutrient sensitivity parameter η due to the symmetric experimental design (i.e. same number of high probability blocks for both options), but nutrient intake increased with higher nutrient sensitivity from nutrient-indiscriminate choices (50% nutrient intake) to nutrient-exclusive choices (100% nutrient intake). The animal obtained higher reward intake due to faster learning driven by the higher nutrient content in the high-probability option. However, the nutrient-sensitive animal was reluctant to switch to low-nutrient choices after probability reversal even if the high-nutrient option was now associated with a lower reward probability. The gain and loss of reward intake would cancel out in a symmetric experimental design as in (A), but the nutrient intake would always increase irrespective of the task structure.

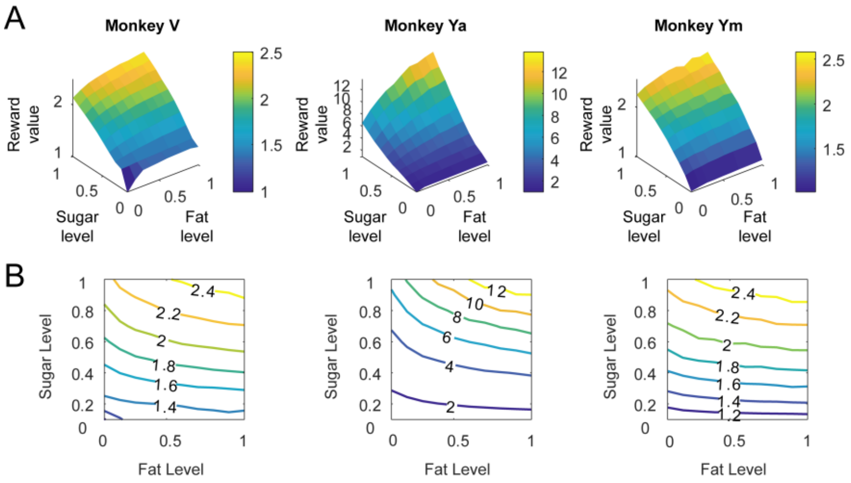

Fig S10. Indifference maps simulated based on the monkeys' nutrient-value functions. (A) Three-dimensional indifference maps constructed by using each animal's regression-derived nutrient-value function to simulate choices for different fat-sugar combinations. Reward values increased continuously with both fat and sugar content with higher gradients for sugar content. **(B)** Two-dimensional iso-contour plots illustrate indifference curves with higher value gradients for sugar content compared to fat content.

Table S1. Nutrient content and texture parameters of liquid food rewards

	Nutrient rewards	Peach flavor				Blackcurrant flavor				Water
		LFLS	HFLS	LFHS	HFHS	LFLS	HFLS	LFHS	HFHS	
Recipe	Peach juice (mL)	30	30	30	30	0	0	0	0	NA
	Blackcurrant juice (mL)	0	0	0	0	30	30	30	30	
	Skimmed milk (mL)	270	0	270	0	270	0	270	0	
	Whole milk (mL)	0	270	0	270	0	270	0	270	
	Caster sugar (g)	0	0.81	20.4	21.21	0	0.81	20.4	21.21	
	Total (mL)	300	300	300	300	300	300	300	300	
Nutrient content (per 100 mL)	Calorie (kcal)	33.5	60.7	60.7	87.9	43.9	71.1	71.1	98.3	0
	Fat (g)	0.45	3.33	0.45	3.33	0.45	3.33	0.45	3.33	0
	Sugar (g)	4.50	4.50	11.30	11.30	6.80	6.80	13.60	13.60	0
	Protein (g)	3.24	3.24	3.24	3.24	3.24	3.24	3.24	3.24	0
	Salt (g)	0.11	0.10	0.11	0.10	0.14	0.13	0.14	0.13	0
	Sugar/fat (kcal/kcal)	4.444	0.601	11.161	1.508	6.716	0.908	13.432	1.815	NA
Texture parameter	Viscosity (19ºC) (cP)*	1.82	4.37	2.00	7.32	1.78	2.18	2.16	2.59	0.99
	Normalized CSF**	0.753	0.638	0.679	0.613	0.653	0.606	0.639	0.574	1.000

* cP = centipoise = mPa*s

**normalized CSF = coefficient of sliding friction normalized with water (CSF_{water} = 1.000)

Table S2. Regression models and performance

Basic regressors = Intercept + First + Spout + RM	AIC	BIC	Loglikelihood	Pseudo-R2
**Monkey V – Peach flavor**	N = 7,718 trials (19 sessions)			
Nutrient model = RM model + Fat + Sugar + Sugar × Fat	4292.342	4535.638	-2111.171	0.605
Basic regressors + Fat + Sugar + Trial × Fat + Trial × Sugar	4061.487	4318.685	-1993.743	0.627
Intercept + First + Spout + Energy	4534.803	4632.121	-2253.402	0.579
Basic regressors + Fat + Sugar + Sugar × Fat + Visc	4304.959	4610.817	-2108.480	0.606
Basic regressors + Fat + Sugar + Sugar ×Fat + CSF	4304.959	4610.817	-2108.480	0.606
Basic regressors + Sugar + Visc + CSF	4291.492	4534.787	-2110.746	0.605
Nutrient model + Sugar × SugarHist 1-10 + Fat × FatHist 1-10 + Fat × SugarHist 1-10 + Sugar × FatHist 1-10	4000.493	4521.841	-1925.247	0.640
**Monkey Ya – Peach flavor**	N = 9,449 trials (23 sessions)			
Nutrient model = RM model + Fat + Sugar + Sugar × Fat	2896.097	3146.476	-1413.049	0.784
Basic regressors + Fat + Sugar + Trial × Fat + Trial × Sugar	2691.821	2956.506	-1308.910	0.800
Intercept + First + Spout + Energy	2906.902	3007.054	-1439.451	0.780
Basic regressors + Fat + Sugar + Sugar × Fat + Visc	2913.025	3227.786	-1412.512	0.784
Basic regressors + Fat + Sugar + Sugar × Fat + CSF	2913.025	3227.786	-1412.512	0.784
Basic regressors + Sugar + Visc + CSF	2908.045	3158.423	-1419.022	0.783
Nutrient model + Sugar × SugarHist 1-10 + Fat × FatHist 1-10 + Fat × SugarHist 1-10 + Sugar ×FatHist 1-10	2735.077	3271.602	-1292.538	0.803
**Monkey Ym – Peach flavor**	N = 7,033 trials (18 sessions)			
Nutrient model = Basic regressors + Fat + Sugar + Sugar × Fat	3550.377	3790.420	-1740.189	0.642
Basic regressors + Fat + Sugar + Trial × Fat + Trial × Sugar	3539.350	3793.110	-1732.675	0.644
Intercept + First + Spout + Energy	4224.665	4320.682	-2098.332	0.569
Basic regressors + Fat + Sugar + Sugar × Fat + Visc	3567.062	3868.830	-1739.531	0.643
Basic regressors + Fat + Sugar + Sugar × Fat + CSF	3567.062	3868.830	-1739.531	0.643
Basic regressors + Sugar + Visc + CSF	3555.661	3795.704	-1742.831	0.642
Nutrient model + Sugar × SugarHist 1-10 + Fat × FatHist 1-10 + Fat × SugarHist 1-10 + Sugar × FatHist 1-10	3569.879	4084.257	-1709.939	0.649
**Monkey V – Blackcurrant flavor**	N = 9,169 trials (20 sessions)			
Nutrient model = Basic regressors + Fat + Sugar + Sugar × Fat	4187.948	4437.273	-2058.974	0.676
Basic regressors + Fat + Sugar + Trial × Fat + Trial × Sugar	4152.486	4416.059	-2039.243	0.679
Intercept + First + Spout + Energy	4858.781	4958.511	-2415.390	0.620
Basic regressors + Fat + Sugar + Sugar × Fat + Visc	4198.753	4512.191	-2055.377	0.676
Basic regressors + Fat + Sugar + Sugar × Fat + CSF	4198.753	4512.191	-2055.377	0.676
Basic regressors + Sugar + Visc + CSF	4185.510	4434.836	-2057.755	0.676
Nutrient model + Sugar × SugarHist 1-10 + Fat × FatHist 1-10 + Fat × SugarHist 1-10 + Sugar × FatHist 1-10	4136.576	4670.845	-1993.288	0.686
**Monkey Ya – Blackcurrant flavor**	N = 13,535 trials (29 sessions)			
Nutrient model = Basic regressors + Fat + Sugar + Sugar × Fat	5685.296	5948.252	-2807.648	0.699
Basic regressors + Fat + Sugar + Trial × Fat + Trial × Sugar	5367.902	5645.884	-2646.951	0.716
Intercept + First + Spout + Energy	5965.324	6070.507	-2968.662	0.682
Basic regressors + Fat + Sugar + Sugar × Fat + Visc	5687.228	6017.801	-2799.614	0.700
Basic regressors + Fat + Sugar + Sugar × Fat + CSF	5687.228	6017.801	-2799.614	0.700
Basic regressors + Sugar + Visc + CSF	5691.504	5954.460	-2810.752	0.699
Nutrient model + Sugar × SugarHist 1-10 + Fat × FatHist 1-10 + Fat × SugarHist 1-10 + Sugar × FatHist 1-10	5577.029	6140.507	-2713.515	0.709
**Monkey Ym – Blackcurrant flavor**	N = 8,301 trials (20 sessions)			
Nutrient model = Basic regressors + Fat + Sugar + Sugar × Fat	3980.256	4226.101	-1955.128	0.660
Basic regressors + Fat + Sugar + Trial × Fat + Trial × Sugar	3959.734	4219.627	-1942.867	0.662
Intercept + First + Spout + Energy	4794.873	4893.211	-2383.436	0.586
Basic regressors + Fat + Sugar + Sugar × Fat + Visc	3990.290	4299.352	-1951.145	0.661
Basic regressors + Fat + Sugar + Sugar × Fat + CSF	3990.290	4299.352	-1951.145	0.661
Basic regressors + Sugar + Visc + CSF	3978.154	4223.998	-1954.077	0.660
Nutrient model + Sugar × SugarHist 1-10 + Fat × FatHist 1-10 + Fat × SugarHist 1-10 + Sugar × FatHist 1-10	3996.916	4523.726	-1923.458	0.666

Table S3. Regression table of the nutrient model

Flavor	Monkey	Regressors	Estimate	SE	t-value	DF	p-value
Peach flavor	**Monkey V** N = 7,718 (19 sessions)	Intercept	-0.057185	0.17803	-0.32121	7711	0.74806
		Spout	0.59454	0.27125	2.1919	7711	0.028419
		First	-0.1577	0.1286	-1.2262	7711	0.22015
		Sugar	3.5703	0.23769	15.021	7711	2.7565e-50
		Fat	1.3938	0.10887	12.802	7711	3.8037e-37
		RM	1.9277	0.24905	7.7405	7711	1.1165e-14
		Sugar × Fat	-0.46566	0.3393	-1.3724	7711	0.16997
	**Monkey Ya** N = 9,449 (23 sessions)	Intercept	0.24861	0.37993	0.65437	9442	0.51289
		Spout	0.53655	0.66735	0.804	9442	0.42142
		First	-0.4	0.26778	-1.4938	9442	0.13527
		Sugar	5.7386	0.42211	13.595	9442	1.058e-41
		Fat	3.2958	0.37169	8.8672	9442	8.867e-19
		RM	1.7922	0.24199	7.4062	9442	1.4112e-13
		Sugar × Fat	-4.0704	0.55829	-7.2907	9442	3.3306e-13
	**Monkey Ym** N = 7,033 (18 sessions)	Intercept	-0.26553	0.15121	-1.756	7026	0.079127
		Spout	1.379	0.20946	6.5833	7026	4.933e-11
		First	-0.18599	0.12168	-1.5285	7026	0.12643
		Sugar	1.522	0.27151	5.6055	7026	2.1552e-08
		Fat	0.55991	0.18927	2.9583	7026	0.0031036
		RM	5.1763	0.2607	19.855	7026	2.1128e-85
		Sugar × Fat	-0.73119	0.49144	-1.4879	7026	0.13683
Blackcurrant flavor	**Monkey V** N = 9,169 (20 sessions)	Intercept	1.2403	0.1342	9.2425	9162	2.9489e-20
		Spout	-2.0985	0.35247	-5.9537	9162	2.7179e-09
		First	-0.0056799	0.27592	-0.020585	9162	0.98358
		Sugar	0.70642	0.18008	3.9228	9162	8.8167e-05
		Fat	0.62955	0.12074	5.2139	9162	1.8891e-07
		RM	5.8851	0.47731	12.33	9162	1.1781e-34
		Sugar × Fat	5.9808	0.41146	14.536	9162	2.4208e-47
	**Monkey Ya** N = 13,535 (29 sessions)	Intercept	0.76681	0.20232	3.79	13528	0.00015131
		Spout	-0.49087	0.39785	-1.2338	13528	0.2173
		First	-0.43209	0.18021	-2.3977	13528	0.016513
		Sugar	4.0922	0.29238	13.996	13528	3.3486e-44
		Fat	2.095	0.42659	4.9109	13528	9.1697e-07
		RM	2.2328	0.31717	7.0398	13528	2.018e-12
		Sugar × Fat	-1.7988	0.58492	-3.0754	13528	0.0021066
	**Monkey Ym** N = 8,301 (20 sessions)	Intercept	-0.81857	0.44558	-1.8371	8294	0.066231
		Spout	2.5297	0.745	3.3956	8294	0.00068797
		First	-0.53788	0.18881	-2.8487	8294	0.0044
		Sugar	1.0788	0.36205	2.9796	8294	0.0028947
		Fat	-0.37921	0.239	-1.5866	8294	0.11263
		RM	5.8858	0.34629	16.997	8294	1.0316e-63
		Sugar × Fat	0.21265	0.39036	0.54476	8294	0.58593

SI References

1. Wood FW (1968) Psychophysical studies on the consistency of liquid foods. *SCI Monograph* 27:40-49.
2. Ogawa H, Ito S, & Nomura T (1989) Oral cavity representation at the frontal operculum of macaque monkeys. *Neurosci Res* 6(4):283-298.
3. Toda T & Taoka M (2002) Hierarchical somesthetic processing of tongue inputs in the postcentral somatosensory cortex of conscious macaque monkeys. *Exp Brain Res* 147(2):243-251.
4. Dresselhuis DM, de Hoog EHA, Stuart MAC, & van Aken GA (2008) Application of oral tissue in tribological measurements in an emulsion perception context. *Food Hydrocolloid* 22(2):323-335.
5. Chojnicka-Paszun A, de Jongh HHJ, & de Kruif CG (2012) Sensory perception and lubrication properties of milk: Influence of fat content. *Int Dairy J* 26(1):15-22.
6. Shewan HM, Pradal C, & Stokes JR (2020) Tribology and its growing use toward the study of food oral processing and sensory perception. *J Texture Stud* 51(1):7-22.
7. Dresselhuis DM, de Hoog EHA, Stuart MAC, Vingerhoeds MH, & van Aken GA (2008) The occurrence of in-mouth coalescence of emulsion droplets in relation to perception of fat. *Food Hydrocolloid* 22(6):1170-1183.
8. Stokes JR, Boehm MW, & Baier SK (2013) Oral processing, texture and mouthfeel: From rheology to tribology and beyond. *Curr Opin Colloid In* 18(4):349-359.
9. Baron RM & Kenny DA (1986) The Moderator Mediator Variable Distinction in Social Psychological-Research - Conceptual, Strategic, and Statistical Considerations. *J Personality Soc Psychol* 51(6):1173-1182.
10. Bollen KA & Noble MD (2011) Structural equation models and the quantification of behavior. *Proc Natl Acad Sci USA* 108 Suppl 3:15639-15646.
11. Bollen KA & Stine R (1990) Direct and Indirect Effects: Classical and Bootstrap Estimates of Variability. *Sociological Methodology* 20:115-140.
12. Shrout PE & Bolger N (2002) Mediation in experimental and nonexperimental studies: new procedures and recommendations. *Psychol Methods* 7(4):422-445.
13. Raubenheimer D (2011) Toward a quantitative nutritional ecology: the right-angled mixture triangle. *Ecol Monogr* 81(3):407-427.
14. Raubenheimer D, Machovsky-Capuska GE, Chapman CA, & Rothman JM (2015) Geometry of nutrition in field studies: an illustration using wild primates. *Oecologia* 177(1):223-234.
15. Committee on Animal Nutrition AHCo, Nonhuman Primate Nutrition NR, & Council (2003) *Nutrient Requirements of Nonhuman Primates: Second Revised Edition* (The National Academies Press, Washington, D.C.).
16. Hinde K & Milligan LA (2011) Primate Milk: Proximate Mechanisms and Ultimate Perspectives. *Evol Anthropol* 20(1):9-23.
17. Chau BK*, et al.* (2015) Contrasting Roles for Orbitofrontal Cortex and Amygdala in Credit Assignment and Learning in Macaques. *Neuron* 87(5):1106-1118.
18. Grabenhorst F, Baez-Mendoza R, Genest W, Deco G, & Schultz W (2019) Primate Amygdala Neurons Simulate Decision Processes of Social Partners. *Cell* 177(4):986-998 e915.
19. Hampton AN, Bossaerts P, & O'Doherty JP (2006) The role of the ventromedial prefrontal cortex in abstract state-based inference during decision making in humans. *J Neurosci* 26(32):8360-8367.
20. Sutton RS & Barto AG (1998) *Reinforcement Learning* (MIT Press, Cambridge, MA).
21. Rescorla RA & Wagner AR (1972) A theory of Pavlovian conditioning: Variations in the effectiveness of reinforcement and nonreinforcement. *Classical Conditioning II: Current Research and Theory*, eds Black AH & Prokasy WF (Appleton Century Crofts, New York), pp 64-99.
22. Cobb CW & Douglas PH (1928) A theory of production. *Am Econ Rev* 18:139-165.
23. Varian HL (2010) *Intermediate Microeconomics* (W. W. Norton & Company, Inc., New York).
